# Poly(hydroxy‐oxazolidone) Thermoplastic Elastomers for Safer, Greener and Customizable Blood‐Contacting Medical Devices

**DOI:** 10.1002/adhm.202502670

**Published:** 2025-06-19

**Authors:** Sofia F. Melo, Anna Pierrard, Fréderic Lifrange, Marco Caliari, Céline D'Emal, Margaux Debuisson, Haritz Sardon, Philippe Delvenne, Patrizio Lancellotti, Christophe Detrembleur, Christine Jérôme, Cécile Oury

**Affiliations:** ^1^ GIGA Metabolism & Cardiovascular Biology – Laboratory of Cardiology University of Liège Avenue de l'Hôpital 11, Quartier Hôpital, Building B34 Liège 4000 Belgium; ^2^ Faculty of Medicine University of Liège Avenue Hippocrate 15, Quartier Hôpital Liège 4000 Belgium; ^3^ Center for Education and Research on Macromolecules (CERM) CESAM Research Unit Department of Chemistry University of Liège Allée du 6 août 13, Building B6a Liège 4000 Belgium; ^4^ Department of Pathology University Hospital Center (CHU) of Liège Avenue de l'Hôpital 11, Quartier Hôpital Liège 4000 Belgium; ^5^ POLYMAT and Department of Polymers and Advanced Materials: Physics Chemistry and Technology Faculty of Chemistry University of the Basque Country UPV/EHU Donostia‐San Sebastian 20018 Spain; ^6^ Heart Valve Clinic University Hospital Center (CHU) of Liège Avenue de l'Hôpital 11, Quartier Hôpital Liège 4000 Belgium; ^7^ WEL Research Institute Wavre 1300 Belgium

**Keywords:** 3D printing, electrospinning, hemo/biocompatibility, medical devices, non‐isocyanate polyurethanes, poly(hydroxy‐oxazolidone)s, thermoplastic elastomers

## Abstract

Thermoplastic elastomers (TPEs) of the polyurethane (PU)‐type have broad applications in healthcare. However, these materials have a number of drawbacks. Their synthesis requires the use of toxic isocyanates.  Their hemocompatibility remains insufficient, resulting in high rates of thrombotic complications of most common blood‐contacting devices, which further increases the risk of infection. Here, we report the facile, up‐scalable preparation of a greener non‐isocyanate polyurethane (NIPU) TPE, poly(hydroxy‐oxazolidone) (PHOx). We show that PHOx can be processed by multiple relevant manufacturing techniques, i.e., hot pressing, injection‐molding, electrospinning, and additive manufacturing. In vitro hemocompatibility tests with human blood demonstrate better performance than a conventional medical grade PU. PHOx triggers less contact phase activation of coagulation, less plasma protein adsorption and less platelet adhesion than PU. The adhesion of *Staphylococcus epidermidis* is also reduced in the first 2 hours of contact as compared to PU. PHOx is neither hemolytic nor cytotoxic upon indirect or direct contact with endothelial cells or fibroblasts. Additionally, subcutaneous implantation of PHOx in rabbits for one and four weeks confirms in vivo biocompatibility and no material degradation. PHOx is therefore a highly valuable biomaterial and a potential isocyanate‐free alternative to conventional PU‐based TPEs for manufacturing customizable blood‐contacting devices with improved hemocompatibility.

## Graphical abstract

A poly(hydroxy‐oxazolidone) (PHOx) is synthesized from CO_2_‐based precursors, enabling the up‐cycling of this waste material. After synthesis, PHOx shows thermoplastic properties, and can therefore be processed by many temperature‐dependent techniques. PHOx is hemocompatible, anti‐adhesive, and biocompatible, which is demonstrated in vitro and in vivo. In some of the performed analysis, PHOx outperforms medical grade polyurethane.

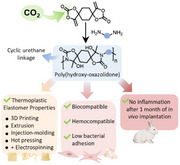

## 1 Introduction

Thermoplastic elastomers (TPEs) are highly desirable for several medical and healthcare applications due to their flexibility/elasticity and processability.^[^ ^1^, ^2^, ^3^ ^]^ TPEs, in particular thermoplastic polyurethanes (TPUs), are used in the form of sutures, coatings, connectors, catheters, leads, tubing, or scaffolds.^[^ ^4^, ^5^, ^6^ ^]^ Their use for the manufacturing of implantable medical devices (MDs) is considerably challenging since the materials must comply to strict safety and performance criteria with a critical impact on patient's outcomes. Materials compliance is particularly challenging for devices that come in contact with blood circulation,^[^ ^7^, ^8^, ^9^ ^]^ such as vascular stents, vascular grafts, intravascular catheters, pacemaker leads, or artificial heart valves. Upon contact with blood, foreign materials indeed trigger a rapid thrombotic response initiated by plasma protein adsorption, platelet adhesion and activation, and contact phase activation of the coagulation cascade.^[^ ^7^, ^10^ ^]^ The surface of MDs can also be colonized by bacteria or fungi during implantation, which can lead to severe bloodstream infections with poor patient's outcome.^[^ ^11^, ^12^, ^13^, ^14^ ^]^ The attempt of treating patients with anti‐thrombotic agents or antibiotics is rarely effective, and the removal of the MD is often the sole solution when complications occur.^[^ ^7^ ^]^ Therefore, when it comes to MD implantation, prevention is preferred. Such preventive approaches include the use of hemocompatible materials^[^ ^15^ ^]^ that would ideally be less prone to bacterial colonization. Notably, since thrombosis and infection are inter‐related processes, improving materials hemocompatibility will automatically reduce infectious complications.^[^ ^16^, ^17^ ^]^ To date, the hemocompatibility of TPE‐made blood‐contacting devices is insufficient. Despite systemic anticoagulation (e.g., heparin), administration of antithrombotic regimens, and/or TPE modifications (e.g., surface functionalization or coatings),^[^ ^18^, ^19^, ^20^ ^]^ high rates of MD‐related thrombotic and infectious complications still occur.^[^ ^21^ ^]^ There is therefore an urgent need for improved materials in the field of implantable blood‐contacting devices. For instance, intravascular catheters are among the most widely implanted MDs for which there is such a need.^[^ ^22^, ^23^ ^]^ They are usually made of polyurethane (PU)‐based TPE, although other TPEs (e.g., TPE block copolymers based on nylon, polyester, or polystyrene) or non‐TPE medical grade polymers (e.g., silicon rubber, polytetrafluoroethylene) can also be found.^[^ ^24^ ^]^ Fibrous PU‐based TPE meshes, typically produced by electrospinning, have applications as scaffolds or tissue‐engineered vascular grafts.^[^ ^25^, ^26^ ^]^

Although widely used, PUs are synthesized from highly toxic isocyanate precursors. Non‐isocyanate polyurethanes (NIPUs) are greener alternatives to PU that substitute the isocyanate reagents for less toxic ones, such as CO_2_‐ and/or bio‐sourced precursors.^[^ ^27^, ^28^, ^29^, ^30^, ^31^, ^32^ ^]^ The first NIPUs developed for biomedical applications have shown promising results, including in tissue engineering,^[^ ^26^, ^33^, ^34^, ^35^, ^36^, ^37^ ^]^ drug delivery,^[^ ^38^ ^]^ antibacterial activity,^[^ ^39^, ^40^, ^41^ ^]^ or blood‐contacting applications.^[^ ^42^, ^43^, ^44^, ^45^ ^]^ Amongst the various NIPU synthesis routes reported, the polyaddition of CO_2_‐based cyclic carbonates bearing exovinylene groups with diamines has recently gained particular interest due to the ease of implementation under mild and catalyst‐free conditions.^[^ ^46^, ^47^, ^48^, ^49^, ^50^, ^51^ ^]^ Moreover, this procedure provides NIPUs, called in this case poly(hydroxy‐oxazolidones) (PHOx)s, bearing rigid and stable cyclic urethane (oxazolidone) linkages that are of particular interest for improving both the mechanical properties and hydrolytic stability of the product compared to conventional NIPU or PU elastomers bearing linear urethane bonds.

**Scheme 1:**
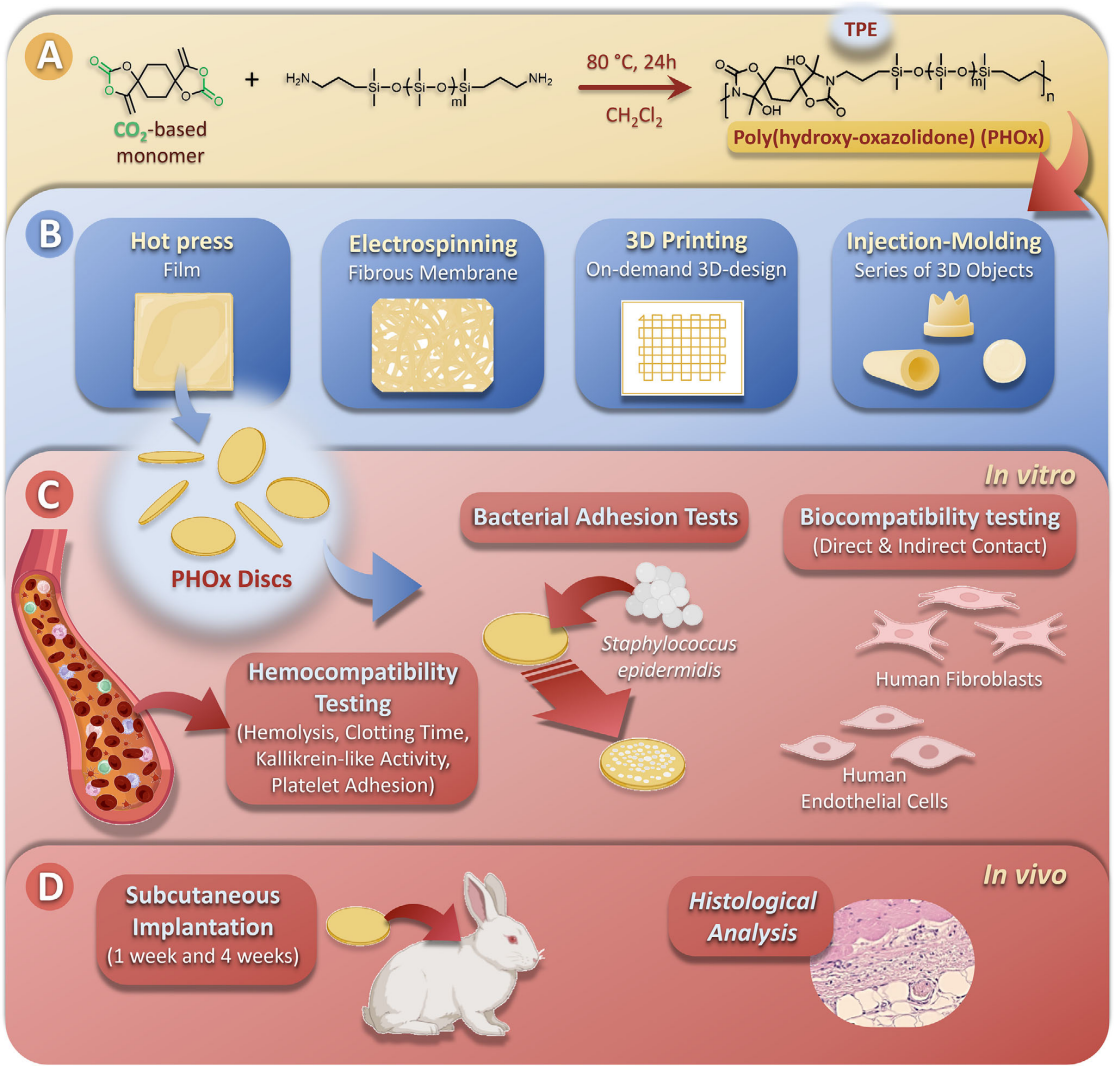
Synthesis of poly(hydroxy‐oxazolidone) (PHOx) thermoplastic elastomer (TPE) from a CO_2_‐based cyclic carbonate (bisαCC) and a polydimethylsiloxane‐based diamine (PDMS‐NH_2_) (A). Different MDs that can be obtained thanks to the easy processing of PHOx TPE by a large range of techniques (B). In vitro biological tests were performed with PHOx discs, namely hemocompatibility assessment, bacterial adhesion tests, and biocompatibility with human fibroblasts and endothelial cells (C). In vivo subcutaneous implantation of PHOx discs in rabbits for periods of 1 and 4 weeks, followed by histological analysis of the tissues (D).

In this work (**Scheme** **1**), we developed the first thermally processable TPE of the poly(hydroxy‐oxazolidone) (PHOx)‐type that alternates soft polydimethylsiloxane (PDMS) segments with rigid hydroxy‐oxazolidone moieties. This novel TPE is obtained by copolymerizing PDMS end‐functionalized by amine groups with a CO_2_‐based bis(alkylidene cyclic carbonate) under catalyst‐free conditions, which has never been achieved before. The physico‐chemical properties of this material were evaluated and demonstrated its huge potential as an alternative to PU‐based TPEs for manufacturing blood‐contacting MDs. PHOx hemocompatibility assessed with human blood was superior to a medical grade PU TPE used as a reference, as PHOx promoted less plasma proteins and platelet adhesion, while triggering less bacteria adhesion in the first 2 h. PHOx was not cytotoxic toward human umbilical vein endothelial cells (HUVEC) and human foreskin fibroblasts (HFF‐1) after indirect and direct contact with the polymer for up to 7 days. Furthermore, the PHOx biocompatibility was confirmed in vivo upon subcutaneous implantation in rabbits for up to 4 weeks. We also demonstrated that this novel TPE could be processed by multiple manufacturing techniques such as hot pressing, injection‐molding, electrospinning, as well as extrusion‐based additive manufacturing, that are all relevant for preparing various biomaterial devices, opening many perspectives in the field.

## 2 Results and Discussion

### 2.1 Synthesis and Characterization of PHOx

A segmented silicone poly(hydroxy‐oxazolidone) (PHOx) was prepared by the step‐growth polyaddition of a commercially available polydimethylsiloxane‐based diamine (PDMS‐NH_2_, poly(dimethylsiloxane) bis(3‐aminopropyl) terminated (Mn = 2,500 g mol^−1^, liquid) with a solid bis(cyclic carbonate) monomer bearing a rigid cyclohexyl spacer (bisαCC) solubilized in CH_2_Cl_2_ (Scheme 1A). This monomer was produced by the catalyzed coupling of CO_2_ to a bis(propargylic alcohol) (1,4‐diethynylcyclohexane‐1,4‐diol) (see Supporting Information for details).^[^ ^49^, ^50^ ^]^ After reaction for 24 h at 80 °C followed by solvent evaporation and cooling to room temperature, an elastic (rubbery) material is obtained. Its chemical structure was first analyzed by ^1^H‐NMR (Figure S1, Supporting Information) and FTIR‐ATR (Figure S2, Supporting Information) spectroscopies which both confirmed the structure of PHOx. An apparent M_n_ of 26,000 g mol^−1^ was determined by gel permeation chromatography (GPC) (Figure S3, Supporting Information) (see Supporting Information for discussion).

The presence of the hydroxyl groups generated beside the urethane bonds on the PHOx structure should bring hydrophilicity to this purposely hydrophobic material based on PDMS segments. Therefore, equilibrium water absorption (EWA) measurements were conducted on the PHOx and evidenced a limited impact of these ‐OH groups as the water absorption remains below 6% (**Table** **1**). Such low swelling in water is particularly desirable and appropriate for biomaterials intended for implantation to avoid excessive volume changes of implants/prostheses after implantation in hydrated environments, so as reduction of the mechanical properties. In accordance, the surface hydrophobicity of PHOx was also confirmed by contact angle measurements on a flat PHOx sheet, which showed a high value of 105° close to data reported for silicones (≈110°)^[^ ^52^, ^53^ ^]^ (Table 1).

**Table 1:** Physico‐chemical and mechanical properties of the PHOx.

Sample	EWA [%]	Water Contact Angle [°]	Mechanical Properties [dry]			Mechanical Properties [wet]			T_d,10%_ [°C]
			E [MPa]	σ [MPa]	ε [%]	E [MPa]	σ [MPa]	ε [%]	
PHOx	5.9 ± 1.5	105 ± 1	2.5 ± 0.03	0.5 ±0.06	52.6 ± 11.2	1.6 ± 0.01	0.2 ± 0.03	23.9 ± 4.2	437

**Figure 1:**
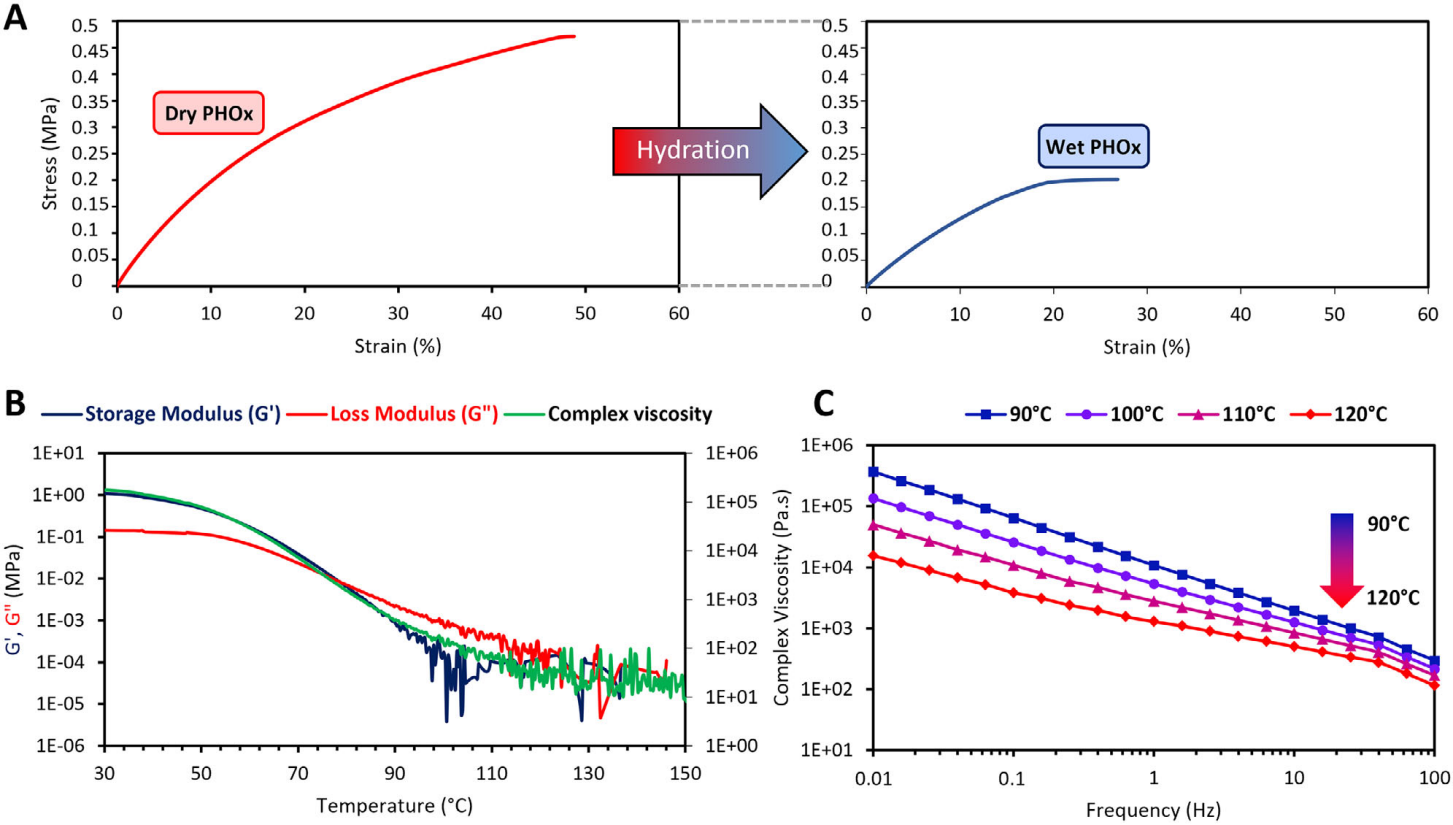
Characterization of the produced PHOx. Example of stress‐strain curves of the PHOx in dry and hydrated states (tensile tests) (A). Temperature sweep experiment showing the decrease of the storage and loss moduli, as well as the complex viscosity with the temperature (B). Complex viscosity measured at different temperatures (frequency sweeps) showing the shear thinning behavior of PHOx (C).

The mechanical properties of this PHOx were then investigated at room temperature, first in the dry state, by tensile tests performed with a dynamic mechanical analyzer (DMA) equipment operating in a non‐oscillating mode (**Figure** **1**A, stress–strain curves of PHOx). The PHOx showed elastomer properties with a Young's Modulus (E) of 2.5 MPa, a stress at break (σ) of 0.5 MPa, and an elongation at break (ε) of 52.6%. Moreover, a strain hysteresis experiment was performed by applying several consecutive tension steps which showed that the polymer went back to its initial position and therefore proved its elastomer behavior (reversibility). The polymer was stretched up to 0.35 MPa (70% of its maximal stress) before being stretched down to 0.00 MPa and relaxed 3 times in a row (Figure S4, Supporting Information). These three consecutive tension cycles showed a very low and stable value of less than 1% of hysteresis (obtained after 5 min of isothermal step and which could even partly come from the error inherent in the machine/experience). This highly attractive elastomer behavior is remarkable and observed for the first time for this type of polymer, namely linear PHOxs. Indeed, the latter are usually either flowing or much stiffer polymers (Young's moduli ≈400–800 MPa)^[^ ^49^, ^54^ ^]^ and therefore not suitable for biomedical applications which require soft materials. This interesting elastomer behavior probably arose from the phase separation between the long and soft PDMS segments and the short and hard (cyclic) hydroxy‐oxazolidone segments, which tend to self‐associate most likely by hydrogen bonds, leading to the cross‐linking of the PDMS segments, similarly to the behavior observed in segmented PUs.^[^ ^55^, ^56^, ^57^, ^58^ ^]^ The mechanical properties of the hydrated samples (after immersion of the samples in MilliQ water for 24 h) were then assessed to examine the impact of hydration on the potential use of PHOx as implants or prostheses, that would be in contact with physiological fluids once implanted. As anticipated, an impact on the PHOx mechanical properties was observed, although relatively minor given the hydrophobic nature of the PDMS segments. Indeed, the values of E, σ, and ε decreased to 1.6 MPa, 0.2 MPa, and 23.9%, respectively, as the result of the water plasticizing effect already reported in the literature for other NIPU materials.^[^ ^42^, ^59^, ^60^, ^61^, ^62^ ^]^ These mechanical characteristics are similar to those of blood vessels since values of E are within the higher range for small‐diameter arteries.^[^ ^63^ ^]^ Healthy coronary arteries, for instance, are reported to have an average modulus of 1.5 MPa, although σ and ε values are slightly superior compared to the ones obtained for PHOx.^[^ ^64^ ^]^ Mechanical properties of PHOx are therefore interesting in the field of small diameter vascular grafts manufacturing, where, for example, poly(ester urethane)‐urea (PEUU)‐containing electrospun fibers with E values ranging from 1.4 to 3.9 MPa are often used.^[^ ^63^, ^65^ ^]^ Another possible application of PHOx is in heart valve substitutes since PHOx displays the properties often found in valve prostheses. Indeed, PHOx mechanical characteristics are close to native valve leaflets (E = 2 MPa, σ = 0.4 MPa, ε = 30%).^[^ ^66^, ^67^ ^]^ Additionally, PHOx could be envisioned as a material for catheter fabrication, although PDMS‐based intravascular catheters have slightly superior mechanical behavior (E > 4 MPa).^[^ ^68^ ^]^

Interestingly, the interactions leading to PDMS crosslinking and responsible for the observed elastomer behavior are thermo‐reversible. Indeed, if the differential scanning calorimetry (DSC) does not evidence neither a glass transition temperature nor a reversible melting transition of the material in the temperature range −80 °C up to 180 °C (Figure S5, Supporting Information), rheological experiment (temperature sweep, Figure 1B), on the other hand, reveals a thermoplastic‐like behavior of PHOx. Indeed, applying a temperature ramp in the range of 30–150 °C leads to the decrease of both the storage (G’) and loss (G’’) moduli which cross at 77 °C. At 30 °C, the storage modulus (1.09 MPa) clearly dominates, the loss modulus being low (0.15 MPa), which is typical of an elastic behavior. These values start to drastically decrease ≈60 °C to reach a viscous behavior above 80 °C. Accordingly, the high complex viscosity of 175,762 Pa.s measured at 30 °C decreases upon heating to reach a quasi‐plateau below 100 Pa.s above 100 °C. It must be noted that the dehydration of hydroxy‐oxazolidone moieties was reported to occur at a temperature ranging from 120 to 140 °C in the solid state,^[^ ^49^, ^51^ ^]^ leading to exocyclic vinylene moieties. This reaction was observed to occur with PHOx above 120 °C, as evidenced by the peak on the non‐reversing heat flow curve in the modulated DSC thermogram (Figure S5, Supporting Information). These observations are also supported by thermogravimetric analysis (TGA) (Figure S6, Supporting Information) which shows a slight weight loss ≈170 °C followed by high thermal stability up to 400 °C (the temperature at 10% weight loss (T_d,10%_) being 437 °C) (Table 1).

As a result, the processing of PHOx TPE should be performed between 80 and 110 °C to avoid any modification of its structure and properties. PHOx is thus well‐suited for conventional thermal‐based manufacturing methods such as extrusion and injection‐molding. The rheological behavior of the PHOx at different frequencies was also analyzed via frequency sweep experiments performed at different temperatures (Figure 1C). Interestingly, PHOx showed a shear thinning behavior along with low complex viscosity values at high frequencies (100 Hz), which indicates their suitability for nozzle‐based 3D printing (see Section 2.2).^[^ ^69^ ^]^

We then evaluated the PHOx stability in vitro through immersion in phosphate‐buffered saline (PBS) over a period of 8 weeks. Visual inspection did not reveal any signs of cracking, or morphological changes, similarly as for PU specimens used as a reference (medical grade Carbothane). The pH of the PBS solutions was then measured weekly (Table S1, Supporting Information) and showed no significant changes over time, being consistently close to 7.4 for both polymers. Another common method to assess degradation is to monitor changes in polymer weight over time. Our results revealed no significant changes over the test period of 8 weeks (Table S2, Supporting Information). The average weight for PU and PHOx samples was maintained, suggesting that the water absorption capacity was kept over time and the polymers were stable. Tensile test experiments were also conducted on samples emerged in PBS for 8 weeks to see if long‐term immersion in an aqueous medium has an impact on the material properties. Interestingly, the latter revealed values of E = 1.4 ± 0.02 MPa, σ = 0.2 ± 0.001 MPa, and ε = 24.1 ± 1.3%, which are very close to those obtained 8 weeks prior. These measurements thereby also demonstrate the stability of PHOx once immersed in an aqueous medium, probably due to the hydrophobic nature of the PDMS segments that mainly constitute the material.

### 2.2 Processing of PHOx TPE

#### 2.2.1 Hot‐Pressing and Injection‐Molding

**Figure 2:**
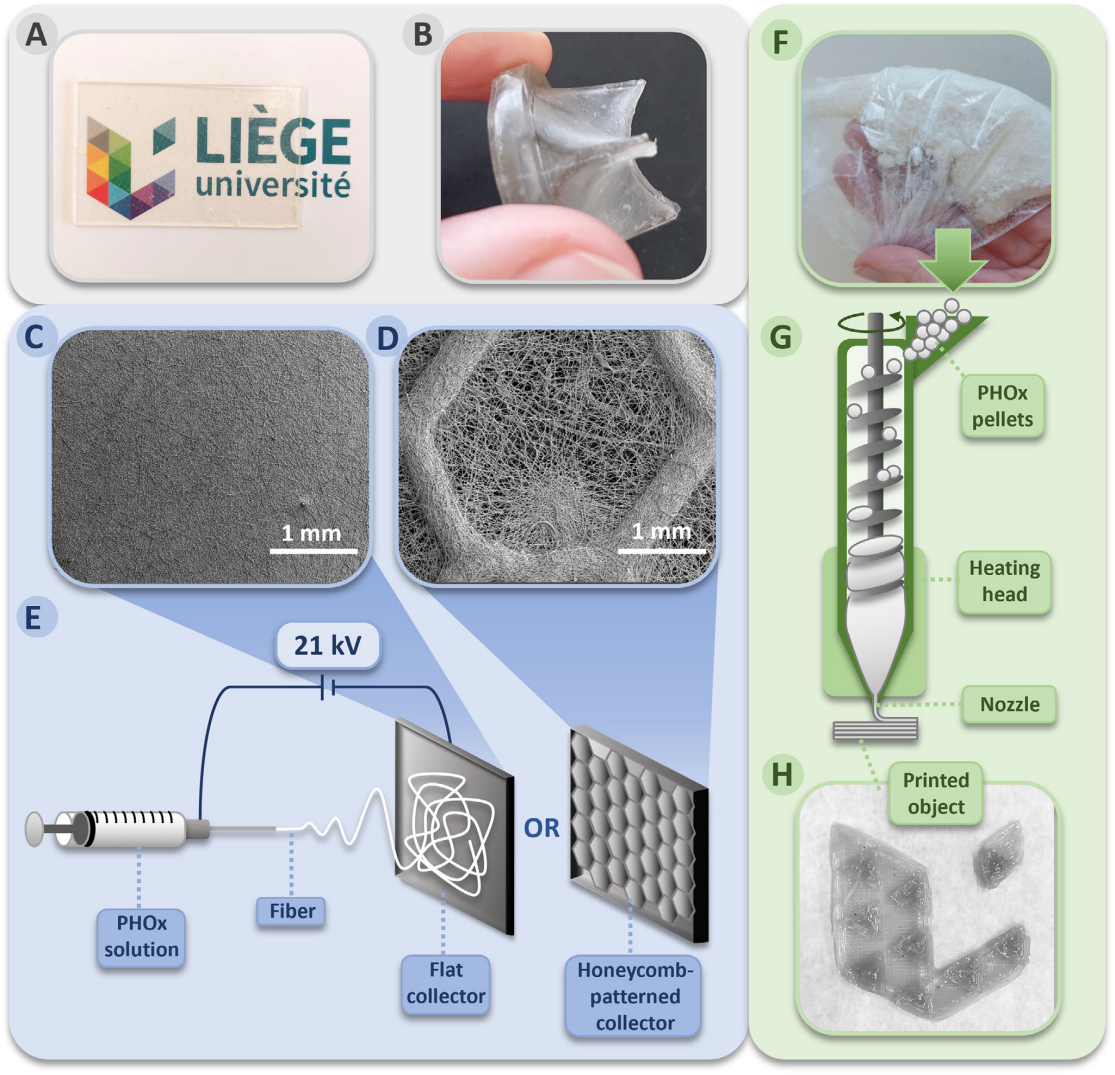
Different PHOx processing techniques. Use of hot‐pressing to produce a film (A). Injection‐molding in a metallic mold to produce a heart valve prosthesis (B). Electrospinning either on a flat sheet aluminum collector, showing a non‐preferential deposition of the fibers (C), or on a honeycomb‐patterned aluminum collector, showing a preferential deposition of the fibers (D). Schematic representation of the electrospinning process (E). Grinded starting polymer used for 3D printing by fused granule extrusion (F). Schematic representation of the thermal‐based additive manufacturing technique (G). 3D printed scaffold (5 × 4 cm) (H).

Considering the thermoplastic behavior of PHOx evidenced by the rheological study, the temperature of 80 °C was chosen as the (re)processing temperature for PHOx samples by using traditional thermal‐based manufacturing methods, i.e., hot pressing and injection‐molding, which allow the production of polymer films or sheets, and various molded objects, respectively. Hot‐pressing (80 °C, 6 metric tons of pressure, 10 min.) provided a 1 mm thick PHOx transparent elastomer film (**Figure** **2**A). The viscous polymer (heated at 80 °C) was also introduced into a pre‐heated stainless‐steel heart valve‐shaped mold,^[^ ^43^ ^]^ which was then cooled down (for 2 h at 6 °C) to produce a heart valve prosthesis prototype after demolding (Figure 2B). These successful processing tests confirmed the (re)processability of PHOx using traditional industrial manufacturing techniques to obtain objects of diverse shapes and did not require high temperatures (here, 80–85 °C), limiting thereby the occurrence of side reactions.

#### 2.2.2 Electrospinning

Electrospinning is an attractive technique used to produce microscale to nanoscale nonwoven fibers starting from polymer solutions. Electrospun fibers are used in many applications,^[^ ^70^ ^]^ and particularly in the biomedical sector for producing scaffolds (e.g., tissue engineering approaches) since fiber mats fabricated with a high surface area‐to‐volume ratio and high porosity are particularly suitable for promoting cell culture, allowing tissue regeneration.^[^ ^71^, ^72^, ^73^ ^]^ During this process, a liquid droplet, located at the outlet of a solution‐filled syringe, is electrified by a high‐voltage power supply to generate a charged jet, which stretches and elongates while the solvent evaporates, to produce fibers that are deposited on a conductive collector (Figure 2E).^[^ ^70^, ^74^ ^]^ Various fibrous PHOx membranes were here produced by electrospinning a solution of PHOx in dichloromethane (0.667 g mL^−1^) for a total electrospinning time of 3 h on three different aluminum collectors. The latter was either a flat aluminum sheet (Figure 2C), or a honeycomb‐patterned collector of different sizes (see Experimental Section for details and Figure 2D). SEM images of the electrospun mats with different magnifications are shown in Figure S7 (Supporting Information). On the flat collector, the fibers were deposited without preferential orientation over the entire surface (Figure S7A, Supporting Information), while on the hollow honeycomb‐shaped collectors, the fibers were deposited preferentially on the edges. Indeed, a greater density of fibers can be seen at the edges than at the centers, which still contain fibers deposited with a non‐preferential orientation (Figure S7B, Supporting Information). The average diameter of the deposited fibers was ≈2.0–2.8 µm for both types of collectors. Their uniform deposition, shape, and diameter demonstrate the successful electrospinning of PHOx and its processing into fiber mats of interest for various applications.^[^ ^70^ ^]^

#### 2.2.3 3D Printing

The 3D printing of this PHOx by a thermal extrusion process was then considered. Additive manufacturing producing devices on‐demand is especially well adapted in the frame of personalized medicine and thus highly developed in this field. A fused granule extrusion (FGE) printer was used as it allows PHOx to be printed below 120 °C, directly from pellets/granules (Figure 2G). This printer was equipped with a nozzle heated at 110 °C and two cooling fans to rapidly cool the printed polymer and avoid heat creep. The bed set was set at 60 °C to increase the adhesion of the first printed layer on the building platform. After optimization, the set parameters (see Experimental Section for details) allowed the successful 3D printing of the University of Liège logo (5 × 4 cm) (Figure 2H). To produce more complex shapes and increase the resolution of future printed parts, the process should still be further optimized, as the low printing temperature of PHOx led to heat creep. Indeed, as seen in Figure 2H and through SEM images (Figure S8A, Supporting Information), the layers of this printed object retained the desired size after printing and stuck well to each other in the Z axis, although a slight under‐extrusion was seen in some limited regions in the X‐Y axis (Figure S8B,C, Supporting Information). This is probably due to the heterogeneous size of the PHOx pellets, which might be overcome by changing the pelletizing procedure. The successful additive manufacturing of the PHOx using this technique allowed the facile and rapid fabrication of customized devices and prototypes. Moreover, compared to traditional manufacturing techniques, FGE requires a low material amount, reduces material waste, and is more rapid and cost‐effective. FGE is even less expensive than fused filament fabrication because the cost of a pellet/granule extruder is significantly lower than that of a filament extruder, given the easier production of pellets.^[^ ^75^, ^76^ ^]^

### 2.3 Biological Evaluation of PHOx

#### 2.3.1 In Vitro Hemocompatibility and Anti‐Fouling Properties Testing

**Figure 3:**
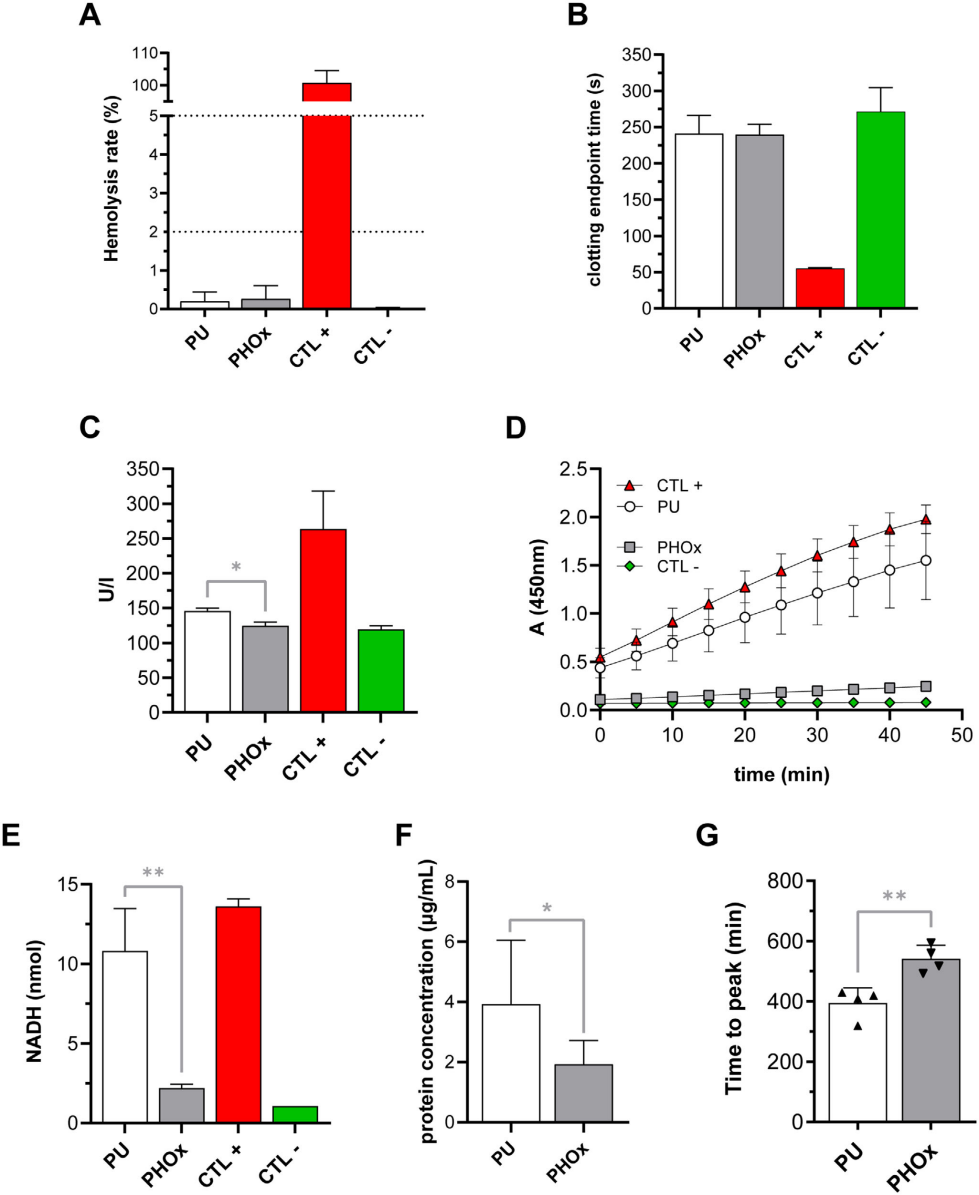
In vitro hemocompatibility and anti‐adhesive properties assessment. Hemolysis rate in RBCs after 3 h of direct contact with PU and PHOx discs, assessed by absorbance measurements in the supernatants. A solution of 1:7 RBCs/distilled water was used as CTL+, while a solution of 1:7 RBCs/PBS was the CTL‐ (A). Clotting endpoint times of PPP after 2 h of direct contact with PU and PHOx discs. Kaolin‐coated glass coverslips were used as CTL+, while PPP alone was used as CTL‐ (B). Plasma kallikrein‐like activity measurements in PPP after 2 h of direct contact with PU and PHOx discs. PPP alone was used as CTL ‐, while PPP incubated with pathromtin (Siemens) was used as CTL +. ^*^ *p* ≤ 0.05, ordinary one‐way ANOVA with Dunnett's multiple comparisons test, *n* = 3 (C). LDH activity of adhered human platelets after 2 h of direct contact with PU and PHOx discs, assessed by a colorimetric assay. CTL+ and CTL‐ are provided with the Sigma‐Aldrich assay kit (D). NADH produced by human platelets adhered on PU and PHOx discs. CTL+ and CTL‐ are provided with the Sigma–Aldrich assay kit. ^**^ *p* ≤ 0.01, ordinary one‐way ANOVA with Dunnett's multiple comparisons test, *n* = 3 (E). Quantification of protein adhesion on PU and PHOx discs after 1 h of incubation with PPP, using colorimetric detection with a micro bicinchoninic acid (BCA) protein assay kit. ^*^ *p* ≤ 0.05, unpaired *t* test, *n* = 6 (F). Measurement of the time needed for the bacterial activity to reach its peak in a population of *S. epidermidis* adhered on PU and PHOx discs. ^**^ *p* ≤ 0.01, unpaired *t* test, *n* = 4 (G).

To characterize the hemocompatibility of PHOx, discs were cut from hot‐press films and sterilized under UV light (germicidal UV‐C lamp, 253.7 nm). We first performed hemolysis tests by incubating the discs with human red blood cells (RBCs) (**Figure** **3**A). Hemolysis rates for PU (medical grade reference) and PHOx were similarly low (below the 2% threshold indicated by the international standards), with values of 0.199 ± 0.238% and 0.261 ± 0.349%, respectively. Clotting times of human platelet‐poor plasma (PPP) were then measured upon incubation with PU and PHOx (Figure 3B). Non‐activated clotting times were equal to 241 ± 25s for PU and 240 ± 14s for PHOx, which did not differ from the value found for control plasma that has not been in contact with the polymers (271 ± 33s). Plasma kallikrein‐like activity was also measured (Figure 3C), which revealed lower activation by PHOx (124.8 ± 5.1 U/l) than by PU (*p* = 0.02). Values found for PHOx did not differ from the activity measured in control plasma (119.6 ± 5.1 U/l). Plasma kallikrein plays an important role in coagulation, acting as a proteolytic activator of factor XII,^[^ ^77^ ^]^ which has critical importance for the initiation of thrombus formation upon contact with foreign materials. The low kallikrein‐like activity measured after incubation with PHOx indicates that the polymer does not support contact phase activation of the coagulation. Complementary to the contact phase, platelet adhesion on surfaces is another key event in materials thrombotic response. We therefore assessed platelet adhesion on the PHOx surface after incubation with platelet‐rich plasma (PRP) (Figure 3D). Adhered platelets were quantified by measuring lactate dehydrogenase (LDH) activity. These assays revealed lower platelet adhesion on PHOx than on PU. LDH activity was close to baseline, hence indicating very low platelet adhesion on PHOx. This result was confirmed by measuring generated reduced nicotinamide adenine dinucleotide (NADH) on the materials surface (Figure 3E), which showed a value of 2.2 ± 0.2 nmol for platelets adhered on PHOx, significantly lower (*p* = 0.001) than the amount produced by samples incubated with PU (10.8 ± 2.7 nmol). To better understand the low platelet adhesion observed on PHOx, we quantified the adhesion of plasma proteins to both polymers, by incubating PU and PHOx with PPP, and measuring protein concentration on the surfaces (Figure 3F). Results revealed a protein concentration of 1.9 ± 0.8 µg mL^−1^ for PHOx samples, significantly lower (*p* = 0.022) than 3.9 ± 2.1 µg mL^−1^ obtained for PU. This protein‐repellent behaviour observed for PHOx might explain, at least partially, the observed low platelet adhesion as compared to PU since plasma protein adsorption is the initial event that leads to platelet adhesion when foreign materials are in contact with blood. Overall, our data showed low thrombogenicity of PHOx, that outperformed medical grade PU. Historically, polycarbonates, polyethers, and polyesters have been widely utilized in segmented PUs intended for biomedical applications.^[^ ^78^ ^]^ To improve the hemocompatibility of PUs, several techniques have been developed, such as surface modifications that increase resistance to protein adsorption and platelet adhesion, while decreasing the hemolysis ratio. PDMS, on the other hand, has been reported to display better hemocompatibility and hydrolytic/oxidative stability, lower toxicity, and higher resistance to protein and platelet adhesion.^[^ ^79^, ^80^, ^81^ ^]^ The fact that PHOx contains soft PDMS segments can probably explain, at least partially, the excellent hemocompatibility of this NIPU.

We then wanted to assess PHOx anti‐fouling property toward bacteria, another crucial criterion when developing polymers for implantable MDs. We investigated the adhesion of a strain of *Staphylococcus epidermidis* (*S. epidermidis*), a biofilm‐forming bacteria that often constitutes a threat for humans (Figure 3G). Bacteria were incubated with the polymers for a pre‐adhesion period of 2 h, after which PU/PHOx discs were gently rinsed to remove non‐adherent bacteria and placed in a microcalorimeter (calScreener), which allows to follow bacterial metabolic activity, which is directly proportional to numbers of adhered bacteria, through real‐time heat flow measurement.^[^ ^82^, ^83^ ^]^ We found that the time needed for *S. epidermidis* to reach the heat flow peak was significantly longer (*p* = 0.005) for bacteria adhered on PHOx (541 ± 45 min), indicating lower numbers of bacteria present after the 2 h pre‐adhesion step. Nevertheless, when the pre‐adhesion period was increased to 24 h, this effect was no longer present (Figure S9, Supporting Information), and no statistically significant differences were found between PU and PHOx. This suggests that an anti‐adhesive effect of PHOx is present in the first minutes after exposure to bacteria, but it fades out after 24 h. Although a long‐term effect would be preferable, the short‐term effect that we observed is still relevant, since it reflects the first hours of contact between the surface and bacteria, which are determinant in the implantation context. This experiment was also performed with Gram – bacteria. In this case, *Pseudomonas aeruginosa* was used, since it is known to cause catheter‐related infections in patients. Again, no differences were observed between PU and PHOx (Figure S10, Supporting Information), neither among the adhered bacteria nor the planktonic ones. We therefore conclude that, after a 24‐h challenge period, PHOx performs similarly to medical grade PU in terms of Gram + and Gram – bacterial colonization. An inhibition of bacterial adhesion on PHOx could not be anticipated based on the literature, that mostly shows that hydrophilic and/or superhydrophobic surfaces inhibit bacterial adhesion. Our PHOx is hydrophobic (contact angle of 105°). Data from longer challenge periods for bacterial adhesion assays are consistent with the fact that PHOx does not potently inhibit bacterial adhesion on the long term. Only early adhesion is decreased as compared to medical PU.

#### 2.3.2 In Vitro Biocompatibility: Interaction with Fibroblasts and Endothelial Cells

**Figure 4:**
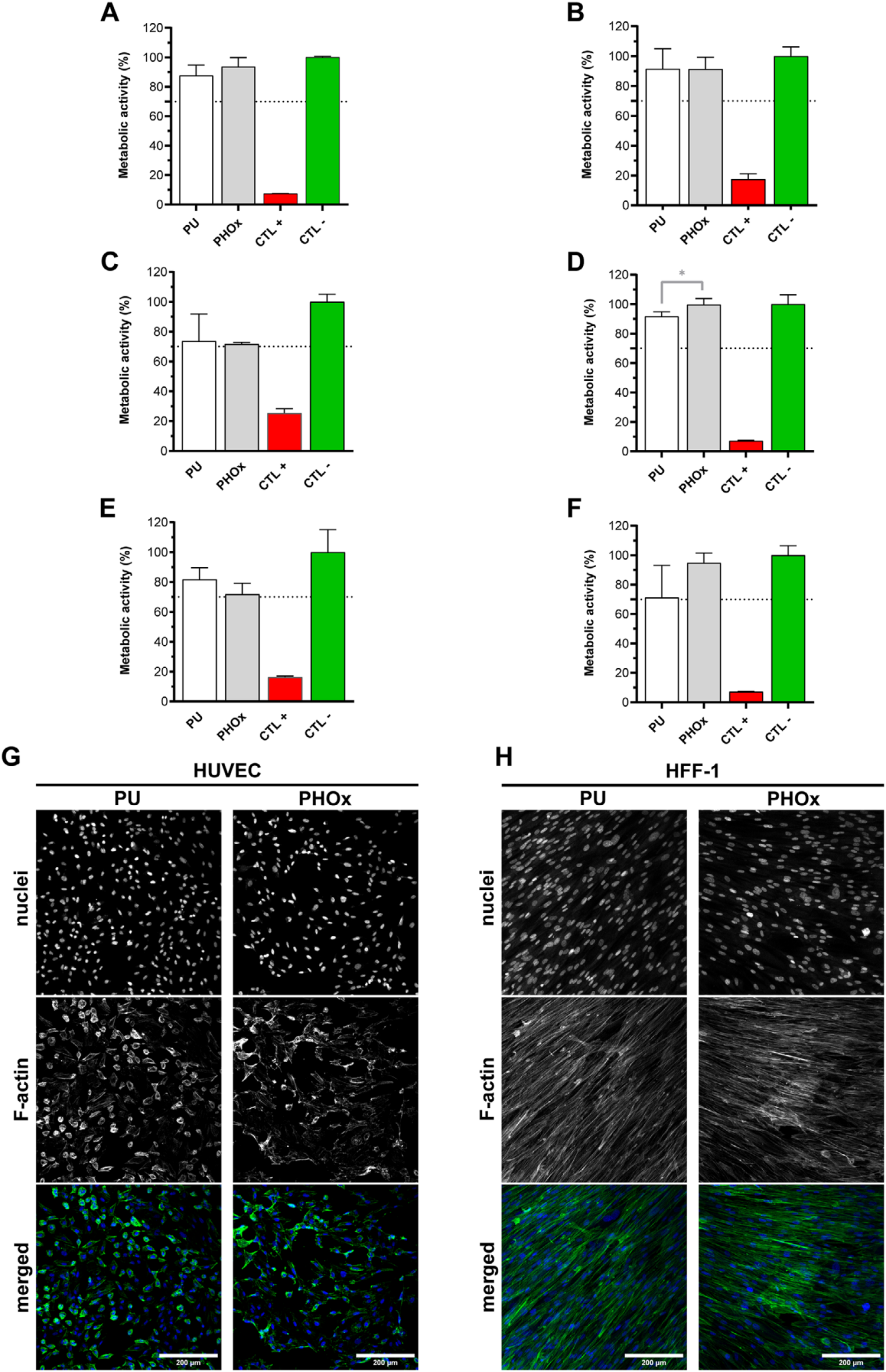
In vitro biocompatibility assessment. Metabolic activity of HUVEC (A) and HFF‐1 (B) after 24 h of indirect contact with PU and PHOx. Metabolic activity of HUVEC (C) and HFF‐1 (D) after 24 h of direct contact with PU and PHOx. ^*^ *p* ≤ 0.05, ordinary one‐way ANOVA with Tukey's multiple comparisons test, *n* = 4. Metabolic activity of HUVEC (E) and HFF‐1 (F) after 7 days of direct contact with PU and PHOx. Cells incubated with culture media were used as CTL ‐, while cells incubated with a solution of Triton (X‐100) 0.1% v/v were used as CTL +. Morphology of HUVEC (G) and HFF‐1 (H) cells after 7 days of direct contact with PU and PHOx. Images represent projections of at least 50‐µm height z‐stacks. Scale bar: 200 µm.

As recommended in ISO 10 993 for new materials intended to contact the human body, we conducted in vitro biocompatibility assays upon indirect and direct exposure of endothelial cells and fibroblasts to PHOx. Metabolic activity measurements were performed after 24 h of indirect contact (**Figure** **4**A,B), 24 h of direct contact (Figure 4C,D) and 7 days of direct contact (Figure 4E,F), after which the morphology of the cells was also observed (Figure 4G,H). Data showed that in all tests (independently of the type and duration of contact) the metabolic activity of both HUVEC and HFF‐1 exposed to PHOx was always higher than 70% (threshold imposed by the international standards). Moreover, after 24 h of direct contact with PHOx, HFF‐1 cells exhibited metabolic activities that were higher than the ones found for PU (92 ± 3% for PU and 100 ± 4% for PHOx, p = 0.03). Cell morphology was verified after 7 days of direct contact with the polymers for both HUVEC and HFF‐1 (nuclei and F‐actin staining). HUVEC displayed a healthy cobblestone‐like shape, while HFF‐1 exhibited normal spindle‐like morphology, after contact with both PU and PHOx. The ensemble of these assessments ensures that PHOx bearing both hydroxyl groups and oxazolidone moieties, i.e., cyclic urethane bonds in their structure, in contrast to conventional PU that only bearing linear urethane groups, is non‐cytotoxic, revealing great biocompatibility in vitro.

#### 2.3.3 In Vivo Biocompatibility: Subcutaneous Implantation of PHOx in Rabbits

**Figure 5:**
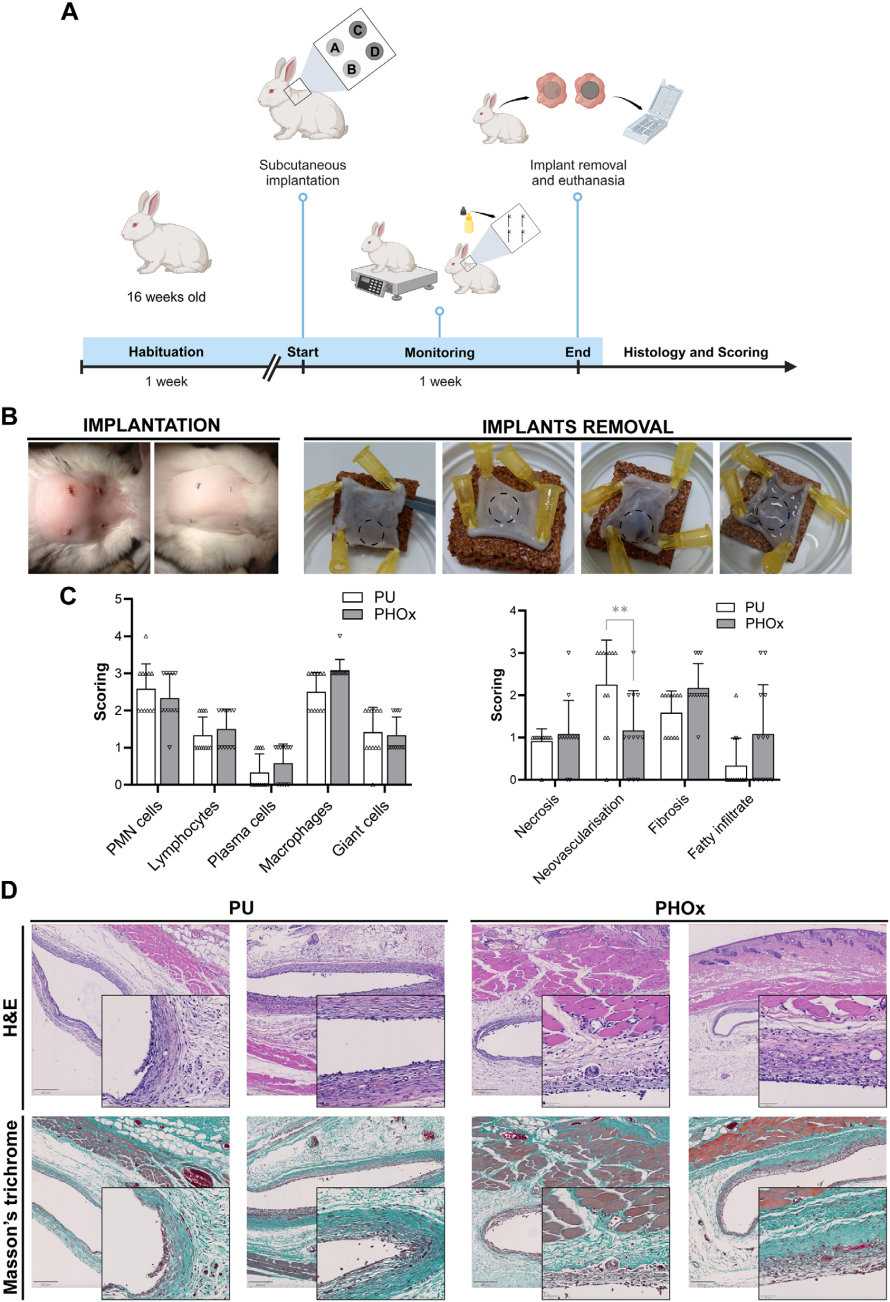
In vivo biocompatibility assessment after 1 week. Scheme of the subcutaneous implantation protocol of PU and PHOx discs in rabbits for 1 week (A). Representative pictures of the skin immediately and 24 h after insertion of 4 implants per animal (left) and implant removal (right) (B). Scoring of different parameters on tissue samples. ^**^ *p* ≤ 0.01, ANOVA with Tukey's multiple comparisons test, *n* = 12 (C). Representative images of histological sections of the tissue surrounding the PU and PHOx implants stained with H&E and Masson's trichrome. Scale bars: 200 µm for overviews, 50 µm for zoomed areas (D).

After verification of in vitro biocompatibility, PHOx discs were tested in vivo by subcutaneous implantation in New Zealand white (NZW) rabbits using medical grade Carbothane as a reference. The first implantation protocol lasted for 1 week (**Figure** **5**A). None of the animals showed abnormal behavior and the wounds resolved similarly for both polymers. Figure 5B shows representative pictures of the 4 sutures performed in each animal (one suture per implant) and the retrieved samples after implant removal. Macroscopically, no apparent differences were observed between PU and PHOx discs, and both were easily separated from the surrounding tissues. All the retrieved implants were intact in terms of shape and diameter. Histopathological scoring (Figure 5C) was performed on hematoxylin‐eosin (H&E) and Masson's trichrome‐stained tissue sections by blinded experts (Figure 5D). The scoring included tissue infiltration with immune cells, which was similar for PU and PHOx, being attributed to the normal early inflammatory response induced by the implantation.

In addition, the scoring evaluated necrosis, neovascularization, fibrosis, and fatty infiltrate. Overall, none or minimal necrosis was observed. Interestingly, the PHOx induced less neovascularization than PU (*p* = 0.0039). Indeed, the excessive formation of new blood vessels represents an undesirable marker in the context of inflammation and adverse foreign body reactions.

**Figure 6:**
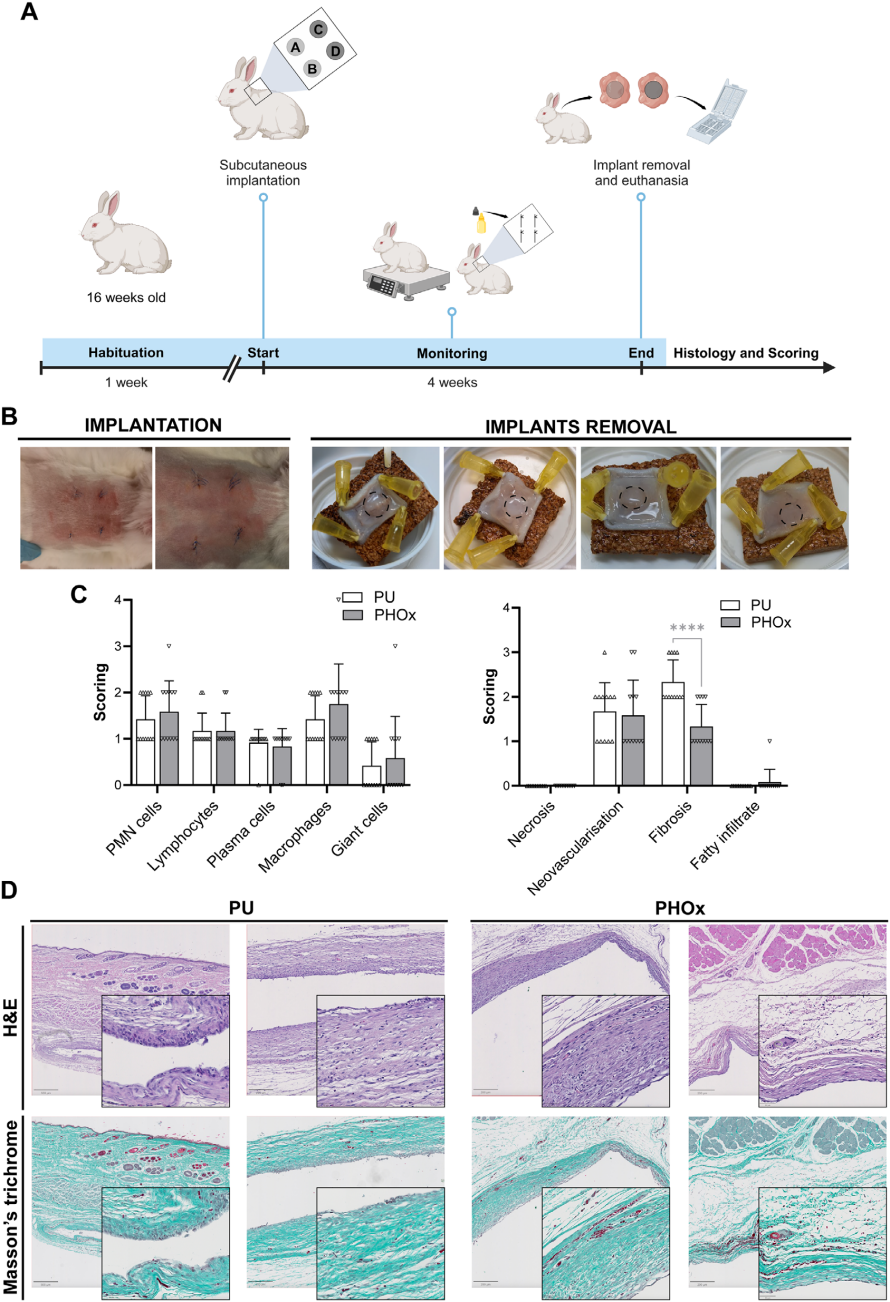
In vivo biocompatibility assessment after 4 weeks. Scheme of the subcutaneous implantation protocol of PU and PHOx discs in rabbits for 4 weeks (A). Representative pictures of the skin 24 and 48 h after insertion of 4 implants per animal (left) and implant removal (right) (B). Scoring of different parameters on tissue samples. ^****^ *p* ≤ 0.001, ANOVA with Tukey's multiple comparisons test, *n* = 12 (C). Representative images of histological sections of the tissue surrounding the PU and PHOx implants stained with H&E and Masson's trichrome. Scale bars: 500 or 200 µm for overviews, 50 µm for zoomed areas (D).

We further investigated PHOx biocompatibility after 4 weeks of implantation (**Figure** **6**A). During follow‐up, none of the animals developed abnormal reactions. Figure 6B shows representative pictures of the 4 sutures performed in each animal (one suture per implant) and the retrieved samples after implant removal. Similarly to what was observed after 1 week of implantation, the implants kept their mechanical and surface characteristics after 4 weeks, with no signs of degradation. Scoring and histology analysis (Figure 6C,D) showed similar levels of infiltrating immune cells for both polymers. No necrosis was observed, with a scoring of 0 for all samples, and almost no fatty infiltration was noticed. Interestingly, PU triggered significantly more fibrosis than PHOx (*p* <0.0001), which was characterized by the presence of larger fibroblasts and denser collagen deposits on PU, stained pink in H&E and blue/green in Masson's trichrome (Figure 6D). Considering that excessive fibrosis may impair MD function and/or cause tissue adherence problems,^[^ ^84^ ^]^ the observation of fewer fibrotic tissue upon PHOx implantation further supports PHOx superiority over medical grade PU reference. Importantly, none of the NIPUs previously described in the literature has ever been tested in vivo for 4 weeks.

## 3 Conclusion

We report a novel isocyanate‐free polyurethane TPE bearing cyclic urethane linkages (oxazolidones), easy to prepare, and which demonstrated remarkable thermo‐mechanical properties similar to those of some native cardiovascular tissues. Its attractive TPE behavior makes it ideal for the facile fabrication of devices of various shapes through a series of industrial manufacturing techniques, such as hot‐pressing, injection‐molding, electrospinning, and even 3D printing. This possibility of cost‐effective and versatile processing of PHOx paves the way to patient‐customizable device production. This PHOx displayed remarkable hemo‐ and biocompatibility that outperformed medical grade PU TPE, the most common material used for manufacturing implantable blood‐contacting MDs such as intravascular catheters. In vitro tests showed that plasma protein adhesion, platelet adhesion, and kallikrein activity were significantly reduced on PHOx surfaces. Moreover, adhesion assays using *S. epidermidis* revealed less bacterial adhesion on PHOx than on medical grade PU after a 2‐h challenge. After 4 weeks of implantation in vivo, PHOx displayed outstanding biocompatibility, without any degradation, and fibrotic scars around PHOx implants were even reduced as compared to PU. Hence, this polymer is a new promising thermally processable material for manufacturing or coating blood‐contacting MDs (eventually patient‐customizable) with improved device performances and patient outcomes.

## 4 Experimental Section

### Synthesis of the Material–Synthesis of the Bis(α‐alkylidene Cyclic Carbonate)

The detailed protocol for the synthesis of bis(α‐alkylidene cyclic carbonate) can be found in the Supporting Information file.

### Synthesis of the Poly(Hydroxy‐Oxazolidone)

The poly(hydroxy‐oxazolidone) (PHOx) was synthesized by adapting Habets’ protocol.^[^ ^50^ ^]^ The bis(α‐alkylidene cyclic carbonate) (bisαCC, synthesis in Supporting Information, 2.016 g, 8 mmol, 1 eq.) was weighed in a 100 mL round flask and magnetically stirred with 8 mL of dry CH_2_Cl_2_. Polydimethylsiloxane bis(3‐aminopropyl) terminated (PDMS‐NH_2_, average Mn = 2,500 g mol^−1^, 20 g, 8 mmol, 1 eq.) was then added under nitrogen atmosphere with a syringe and the solution was stirred at 80 °C for 24 h. The PHOx was then analyzed by ^1^H‐NMR (Figure S1, Supporting Information), FTIR (Figure S2, Supporting Information), and GPC (Figure S3, Supporting Information). GPC in chloroform showed an apparent number‐average molar mass of 26,000 g mol^−1^. The PHOx was then also characterized by DSC (Figure S5, Supporting Information), TGA (Figure S6, Supporting Information), contact angle, equilibrium water absorption, DMA (Figure 1A; Figure S4, Supporting Information), and rheology experiments (Figure 1B,C). The polymer was stored in a desiccator to avoid any humidity absorption prior to any measurement.

PHOx: ^1^H‐NMR (400 MHz, CDCl_3_) δ (ppm) = 3.13 (s, 2H), 2.24 – 1.43 (m, 10H), 1.36 (d, *J* = 8.2 Hz, 3H), 0.46 (s, 2H), 0.07 (s, 108H).

### Processing of the Material–Procedure for Hot Pressing

PHOx films were produced by placing pieces of grinded PHOx in a 1 mm thick mold (metal plate perforated at its center and pressed between 2 Teflon films and 2 full metal plates), using a 4122CE.4010C00 press (Carver) for 10 min at 80 °C and with a pressure of 6 metric tons.

### Procedure for Injection Molding

PHOx was introduced directly after its synthesis (while still hot) into a stainless‐steel heart valve‐shaped mold developed by our team^[^ ^43^ ^]^ previously coated with a polyvinyl alcohol (PVA) release agent to facilitate unmolding. The PHOx‐filled mold was heated for 2 h at 85 °C in an oven and then cooled in the refrigerator for 2 h at 6 °C before opening to unmold the object. The PHOx heart valve was then gently washed with water to remove the PVA release agent.

### Procedure for Electrospinning

Fibrous PHOx membranes were produced by electrospinning a solution of PHOx in dichloromethane (0.667 g mL^−1^) placed in a 10 mL syringe (internal diameter of 13 mm) on an aluminum collector (either flat sheet or honeycomb patterned of either 3 mm hexagonal side and 7 mm thickness or 1.3 mm hexagonal side and 5 mm thickness), using an electrospinning high voltage equipment (ND‐ES Electrospinning). The voltage was initially set at 21 kV and increased by 1 kV every hour; the flow rate was set at 5 µL min^−1^, the distance between the top of the needle and the collector was set at 17 cm, and the total electrospinning duration time was fixed as 3 h. The electrospun mats were then analyzed by scanning electron microscopy (SEM) using an Ultra‐High Resolution Scanning Electron Microscope (TESCAN CLARA).

### Procedure for 3D Printing

PHOx was grinded and then extruded through a twin‐screw extruder at 70 °C and 30 rpm to obtain a homogenous filament. The filament was let to rest for 30 min at room temperature and then cut into pellets using a pelletizer. The resulting pellets were stored in a desiccator to avoid any humidity absorption. Pellets were then charged in an Ender 3 NEO retrofitted with a V4 pellet extruder and a 0.8 mm diameter nozzle. The nozzle was set at 110 °C and the heated build platform (bed set) at 60 °C. A printing speed of 7.5 mm s^−1^ together with an extrusion multiplier of 1.2 was applied. Layers of 0.2 mm were printed. A skirt of 6 lines was used to reach a stable filament deposition. A polypropylene film was employed as substrate as this was reported to adhere better to hydrophobic polymers. To avoid heat creep, two cooling fans were installed: one cooling the pellet hopper, and a second one close to the heated head. SEM observation and measurements of the printed samples were conducted on a Hitachi TM3030Plus microscope.

### Biological Testing of the Material

In all biological tests, medical grade Carbothane PU was used as reference material. Carbothane PU, as well as the synthesized PHOx, were cut into discs and sterilized under UV (germicidal UV‐C lamp, 253.7 nm), by exposing each side 15 min.

### In Vitro Hemocompatibility and Anti‐Fouling Properties Testing

For hemocompatibility tests, blood samples from healthy donors were used. Informed consent was obtained. The study was approved by the Ethics Committee of the University Hospital of Liège, Belgium, and was performed in accordance with EU regulations on the collection and use of samples of human body material for research purposes (Royal Decree and the provisions of the Act concerning biobanks entered into force in November 2018, law 19/12/2008).

### Hemolysis Test (Erythrocytes)

In vitro hemocompatibility was first evaluated through hemolysis testing, using red blood cells (RBCs). The procedure is previously described in detail by the authors^[^ ^42^, ^43^ ^]^ and a summary can be found in the Supporting Information file.

### Coagulation Test (Clotting Time)

For these experiments, platelet‐poor plasma (PPP, CRYOcheck) was used. The procedure is previously described in detail by the authors^[^ ^42^, ^43^ ^]^ and a summary can be found in the Supporting Information file.

### Kallikrein‐Like Activity Measurement

First, the substrate (S‐2302, Chromogenix) was reconstituted in dH_2_O and tris buffer was prepared in‐house, with pH adjusted to 7.8 at 25 °C. PU and PHOx discs were incubated with PPP for 2 h at 37 °C. PPP alone was used as CTL ‐, while PPP incubated with pathromtin (Siemens) was used as CTL +. CaCl_2_, tris buffer, S‐2302, and acetic acid (acid‐stopped method) were added sequentially to the incubated plasma, following the timing and the amounts recommended by Chromogenix. Absorbance values (λ ≈405 nm) were measured and compared to CTL – and CTL +. The following equation was used to calculate plasma kallikrein‐like activity in enzyme activity units: U/l = A x 344, where A is the read absorbance.

### Lactate Dehydrogenase Activity (Platelet Adhesion)

The procedure is previously described in detail by the authors^[^ ^42^, ^43^ ^]^ and a summary can be found in the Supporting Information file.

### Micro Bicinchoninic Acid (BCA) Protein Assay Kit (Protein Quantification)

First, PU and PHOx discs were incubated with PPP for 1 h at 37 °C, under mild agitation (100 rpm). After incubation, the disks were rinsed to remove unbound proteins, and a solution of sodium dodecyl sulfate (SDS) was then used to recover adhered proteins. To quantify the recovered protein concentration, the micro BCA protein assay kit (Thermo Scientific) was used. Bovine serum albumin (BSA) standard ampules (2 mg mL^−1^), provided with the kit, were used to prepare different solutions, at different concentrations, and plot a standard curve. Kit reagents were added following the amount and timing recommended in the user guide. Absorbance values (λ ≈562 nm) were measured, allowing colorimetric detection and quantitation of total protein in each sample.

### Anti‐Adhesive Properties Against Bacteria (2‐h Challenge)

Bacterial adhesion assays were performed using a biofilm‐forming strain of *Staphylococcus epidermidis* (*S. epidermidis*) (ATCC 35 984). PU and PHOx discs were incubated with a bacterial inoculum of 10^5^ CFUs/mL in tryptic soy broth (TSB) for 2 h at 37 °C in static conditions. After the 2‐h adhesion step, polymer discs were rinsed three times with 500 µL of PBS, so unattached or poorly attached bacteria could be removed. The discs with adherent bacteria were transferred to optical grade sterile plastic inserts (calWell, SYMCEL) and 300 µL of fresh TSB was added to each sample. The plastic inserts were then placed inside titanium cups, closed with titanium lids, and the metallic ensemble was incubated in our biocalorimeter (calScreener, SYMCEL) for 24 h under static conditions at 37 °C. Bacterial activity was measured through the detection of the produced heat, a by‐product of biological processes.

### In Vitro Biocompatibility: Interaction with Fibroblasts and Endothelial Cells

In vitro biocompatibility assays were performed using a cell line of human fibroblasts (HFF‐1, ATCC‐SCRC‐1041, RRID: CVCL_3285) and pooled human umbilical vein endothelial cells (HUVEC, Catalog #C2519A, Lot #23TL163040). Cells were tested, confirming no contamination. Cells were grown as previously described in detail by the authors.^[^ ^43^ ^]^ When reaching 90% confluence, cells were rinsed with 5 mL of PBS (37 °C) and detached from culture flasks using 2 mL of Trypsin‐EDTA solution.

### Indirect Contact (Extracts)

The cytocompatibility of PHOx was initially evaluated using the indirect contact assay, previously described in detail by the authors.^[^ ^43^ ^]^ Briefly, cells were incubated for 24 h with extracts of the materials, which were prepared as described in ISO 10993−12:2004. A solution of Triton (X‐100) 0.1% v/v was used as CTL + of cytotoxicity. The culture medium was used as CTL ‐.

### Direct Contact

The cytocompatibility of PHOx was confirmed by direct contact between polymer discs and HFF‐1/HUVEC cells for 24 h and 1 week. Cells were seeded at a density of 10^5^ cells mL^−1^ and kept in culture for up to 1 week at 37 °C with 5% v/v CO_2_ in direct contact with PU and PHOx discs. A solution of Triton (X‐100) 0.1% v/v was used as CTL + of cytotoxicity. The culture medium was used as CTL ‐.

### Measurement of the Metabolic Activity

The metabolic activity of the cells was quantified after 24 h of indirect contact and after 24 h and 1 week of direct contact with the polymers, using a resazurin‐based assay. For that, the culture medium was removed and fresh media containing 10% v/v resazurin was incubated with the cells for 4 h at 37 °C with 5% v/v CO_2_. Metabolized medium was then transferred to black 96‐well plates and the relative fluorescence units (RFUs) were measured (λ_ex_ ≈530 nm, λ_em_ ≈590 nm) using a microplate reader. Results were displayed as percentages calculated relative to the CTL ‐.

### Visualization of Adherent Cells

After 1 week of culture, HFF‐1 and HUVECs were fixed using paraformaldehyde (PFA) 4% w/v in PBS for 15 min, rinsed with PBS, and prepared for fluorescent microscopy. Cells were stained for the identification of deoxyribonucleic acid (DNA) and filamentous actin (F‐actin), using 6‐diamidino‐2‐phenylindole dihydrochloride (DAPI) in a concentration of 3 µg mL^−1^, and phalloidin conjugated with Alexa Fluor 488 in a 1:100 dilution, respectively. Incubation with phalloidin was performed for 1 h in the dark, under mild agitation, while incubation with DAPI lasted for 15 min. Cells were observed with a confocal Nikon A1R hybrid resonant microscope. Representative scanned z‐series of the samples were projected onto a single plane and pseudo‐colored using ImageJ software.

### In Vivo Biocompatibility: Subcutaneous Implantation of PHOx in Rabbits

The animal experiments described were performed after approval of the protocols by the Institutional Animal Care and Use Committee of the University of Liège, Belgium, in agreement with the European Directive 2010/63 on the protection of animals used for scientific purposes. Our Laboratory has the necessary accreditation number (LA1610002), and the experiments are described in file #2198. Sample size determination was based on ISO 10993–6 recommendations and 12 discs of each polymer were implanted for each time point. Two medical grade PU and two PHOx discs were implanted per animal, using therefore a total of 6 animals for each time point of the experiment.

### Animal Model and Housing Conditions

In vivo biocompatibility assays were performed using female New Zealand white (NZW) rabbits, 112–118 days old (Charles River Laboratories, France). Housing conditions were compliant with ISO 10993‐2 requirements. Animals were kept in a temperature and humidity‐controlled environment with standard rabbit chow and tap water ad libitum. Rabbits had a period of acclimation of at least 1 week and were inspected every 24 h.

### Surgery

Surgery was performed according to ISO 10993–6 recommendations, after general anesthesia and in a manner that minimizes trauma at the implant site. After weighting the animals, anesthesia was induced by intramuscular injections of droperidol (0.625 mg kg^−1^), xylazine (5 mg kg^−1^), and ketamine (35 mg kg^−1^) in the hind legs. The hair from the surgical area (dorsal) was removed by shaving, to ensure that the implants or wound surfaces did not come in contact with hair. The exposed area of the skin was disinfected, an incision to create a pocket for each polymer disc was opened, and sterilized PU and PHOx discs were placed subcutaneously, with a spacing of at least 2 cm between different implants. The pockets were closed with at least 2 stitches using a Prolene suture.

### Post‐Surgical Procedures

Rabbits awakening phase was typically shorter than 1 h post‐implantation. Animals were surveilled every 30 min on the day of implantation, and every 24 h in the days after. Buprenorphine (0.05 mg kg^−1^) was injected subcutaneously 24 h after implantation. In case of scratching around the closed wounds, iodopovidone (iso‐Betadine Gel 10%, Mylan) was applied in the irritated skin.

### Euthanasia, Implant Retrieval and Tissue Sample Collection

After 1 or 4 weeks, the animals were anesthetized, euthanized, and implants were removed together with the surrounding tissues. Euthanasia was conducted in anesthetized animals by intracardiac injection of pentobarbital (200 mg kg^−1^). The implants, together with the surrounding tissues, were fixed in a solution of 4% PFA in PBS for 24 h. The implants were then separated from the tissues before histological preparation. Tissues were dehydrated in a solution of 70% ethanol (overnight at room temperature) and then embedded in paraffin wax, sectioned, and stained with hematoxylin‐eosin (H&E) and Masson's trichrome. Histological sections were analyzed by pathology experts and scored.

### Scoring of Inflammation Markers

The local effects were evaluated by a comparison between the tissue histopathological responses caused by PHOx (test specimen) and that caused by PU (used in medical devices with established clinical acceptability), following the ISO 10993–6. Biological response parameters that were analyzed include: the extent of fibrosis/fibrous capsule; the changes in tissue morphology; the number and distribution of polymorphonuclear neutrophilic (PMN) leucocytes, lymphocytes, plasma cells, macrophages, and giant cells; the presence, extent, and type of necrosis; tissue alterations such as neovascularization or fatty infiltration; the material parameters such as fragmentation and/or debris presence. The scoring procedure was based on the ISO 10993–6 recommendations (Annex E, tables E.1 and E.2). Comparison between different inflammation markers in PU or PHOx was performed by ANOVA with Tukey's multiple comparisons test.

### Statistical Analysis

Data was presented as mean value ± SD in all the graphs shown in Figures 3, 4, 5, and 6. The statistical tests and multiple comparisons used to reveal statistically significant differences, as well as the sample sizes (n), varied among the different statistical analysis: ^*^ *p* ≤ 0.05, ordinary one‐way ANOVA with Dunnett's multiple comparisons test, *n* = 3, for Figure 3C; ^**^ *p* ≤ 0.01, ordinary one‐way ANOVA with Dunnett's multiple comparisons test, *n* = 3, for Figure 3E; ^*^ *p* ≤ 0.05, unpaired *t*‐test, *n* = 6, for Figure 3F; ^**^ *p* ≤ 0.01, unpaired *t*‐test, *n* = 4, for Figure 3G; ^*^ *p* ≤ 0.05, ordinary one‐way ANOVA with Tukey's multiple comparisons test, *n* = 4, for Figure 4D; ^**^ *p* ≤ 0.01, ANOVA with Tukey's multiple comparisons test, *n* = 12, for Figure 5C; and ^****^ *p* ≤ 0.001, ANOVA with Tukey's multiple comparisons test, *n* = 12, for Figure 6C. GraphPad Prism was the software used for statistical analysis.

## Conflict of Interest

The authors declare no conflict of interest.

## Author Contributions

S.F.M., A.P., C.J., and C.O. contributed equally to this work. S.F.M. contributed to conceptualization, formal analysis (including in vitro degradation tests, in vitro hemocompatibility and biocompatibility tests, microbiology assays, and in vivo implantation), investigation, and original draft writing. A.P. was involved in conceptualization, formal analysis (synthesis, characterization, and processing techniques), investigation, and original draft writing. F.L. conducted a formal analysis of tissue sample scoring, while M.C. performed a formal analysis related to 3D printing. C.D. and M.D. carried out formal analysis and investigation for in vivo implantation. H.S., P.D., and P.L. provided supervision. C.D., C.J., and C.O. contributed to supervision and were involved in reviewing and editing the manuscript.

## Supporting information



Supporting Information

## Data Availability

The data that support the findings of this study are available from the corresponding author upon reasonable request.
