# Supplementary material for: Poly(hydroxy‐oxazolidone) Thermoplastic Elastomers for Safer, Greener and Customizable Blood‐Contacting Medical Devices
Source: Adv Healthc Mater. 2025 Jun 19;14(23):2502670. doi: 10.1002/adhm.202502670 (PMC12417777; doi:10.1002/adhm.202502670)
Supplement: Supplementary file 1 — Supporting Information [file ADHM-14-0-s001.pdf]

ADVANCED HEALTHCARE MATERIALS

Supporting Information

for *Adv. Healthcare Mater.*, DOI 10.1002/adhm.202502670

Poly(hydroxy-oxazolidone) Thermoplastic Elastomers for Safer, Greener and Customizable Blood-Contacting Medical Devices

Sofia F. Melo, Anna Pierrard, Fréderic Lifrange, Marco Caliari, Céline D'Emal, Margaux Debuisson, Haritz Sardon, Philippe Delvenne, Patrizio Lancellotti, Christophe Detrembleur, Christine Jérôme and Cécile Oury**

SUPPORTING INFORMATION

Poly(hydroxy-oxazolidone) Thermoplastic Elastomers for Safer, Greener and Customizable Blood-contacting Medical Devices

Sofia F. Melo[‡a,b], *Anna Pierrard*[‡c], *Fréderic Lifrange*[d], *Marco Caliari*[c,e], *Céline D'Emal*[a], *Margaux Debuisson*[a], *Haritz Sardon*[e], *Philippe Delvenne*[d], *Patrizio Lancellotti*[a,f], *Christophe Detrembleur*[c,g], *Christine Jérôme*[c,#,]*, *Cécile Oury*[a,#,]*

[‡]Authors contributed equally to this work.

[a]GIGA Metabolism & Cardiovascular Biology - Laboratory of Cardiology, University of Liège, Avenue de l'Hôpital 11, Quartier Hôpital, Building B34, 4000 Liège, Belgium. E-mail: Cecile.Oury@uliege.be

[b]Faculty of Medicine, University of Liège, Avenue Hippocrate 15, Quartier Hôpital, 4000 Liège, Belgium.

[c]Center for Education and Research on Macromolecules (CERM), CESAM Research Unit, Department of Chemistry, University of Liège, Allée du 6 août 13, Building B6a, 4000 Liège, Belgium. E-mail: c.jerome@uliege.be.

[d]Department of Pathology, University Hospital Center (CHU) of Liège, Avenue de l'Hôpital 11, Quartier Hôpital, Liège, 4000, Belgium.

[e]POLYMAT and Department of Polymers and Advanced Materials: Physics, Chemistry and Technology, Faculty of Chemistry, University of the Basque Country UPV/EHU, 20018 Donostia-San Sebastian, Spain.

[f]Heart Valve Clinic, University Hospital Center (CHU) of Liège, Avenue de l'Hôpital 11, Quartier Hôpital, Liège, 4000, Belgium.

[g]WEL Research Institute, 1300 Wavre, Belgium

[#,]*Corresponding authors who contributed equally to this work.

MATERIALS

1,4-Cyclohexanedione (98%, Thermo Scientific Chemicals), acetonitrile (≥99.9%, Fischer Chemicals), ammonium chloride (≥99.5%, Thermo Scientific Chemicals), carbon dioxide (N27), chloroform (≥99.8%, VWR Chemicals), copper(I) iodide (≥99.5, Sigma-Aldrich), dichloromethane (≥98%, VWR Chemicals), diethylether (rectified, VWR Chemicals), ethynylmagnesium bromide solution (0.5 M in THF, Sigma-Aldrich), glacial acetic acid (≥99.9%, VWR Chemicals), magnesium sulfate (≥98%, VWR Chemicals), methanol (≥99.8%, VWR Chemicals), tetrabutylammonium bromide (≥98%, Sigma-Aldrich), tetrahydrofuran (≥99.8%, Fisher Chemicals), poly(dimethylsiloxane) bis(3-aminopropyl) terminated (average Mn = 2,500 g/mol, Sigma-Aldrich and viscosity 20-30 cSt, ABCR), phenol (≥99.8%, Sigma-Aldrich), potassium hydroxide (≥99.9%, Acros Organics), Carbothane™ (Lubrizol), phosphate buffered saline (PBS) (Gibco, Thermo Fisher Scientific), CRYOcheck™ pooled normal plasma (Cryopep), 5-CLOT NaPTT reagent (NODIA), CaCl2 solution 1M (Merck), Chromogenix S-2302 (Werfen), pathromtin (Siemens), triton™ X-100 (Merck), lactate dehydrogenase (LDH) activity assay kit (Sigma-Aldrich), *Staphylococcus epidermidis* (ATCC 35984), *Pseudomonas aeruginosa* (ATCC 15442), tryptic soy broth (TSB), tryptic soy agar (TSA), human fibroblasts HFF-1 (SCRC-1041, ATCC, RRID:CVCL_3285), Dulbecco's modified Eagle's medium (DMEM) (Gibco, Thermo Fisher Scientific), fetal bovine serum (FBS) (Gibco, Thermo Fisher Scientific), penicillin/streptomycin (Pen/Strep) (Biowest), pooled human endothelial cells HUVEC (Catalog #C2519A, Lot #23TL163040), EGM™-2 Endothelial Cell Growth Medium-2 BulletKit™ (Lonza), trypsin-EDTA solution (Gibco, Thermo Fisher Scientific), 10 oxido-7-oxophenoxazin-10-ium-3-olate (resazurin) (Stemcell Technologies), paraformaldehyde (PFA) (Merck), 6-diamidino-2-phenylindole dihydrochloride (DAPI) (Merck), phalloidin Alexa Fluor® 488 (Molecular Probes),

droperidol (Dehydrobenzperidol 2.5 mg/mL, ProsTraKan), xylazine (Proxylaz 2%, Prodivet pharmaceuticals), ketamine (Nimatek 100 mg/mL, Eurovet), buprenorphine (Bupaq 0.3 mg/mL, Richter pharma), iodopovidone (iso-Betadine Gel 10%, Mylan), pentobarbital (Euthasol vet 400 mg/mL, KELA).

CHARACTERIZATION METHODS

Fourier transformed infrared spectroscopy (FTIR). Fourier transformed infrared spectra were collected with a Nicolet IS5 spectrometer (Thermo Fisher Scientific) using a diamond attenuated total reflectance (ATR) device. The spectra were obtained in transmission/ATR mode as a result of 32 spectra in the range 4,000 – 500 cm^{-1}.

Nuclear magnetic resonance spectroscopy (NMR). The samples were prepared by dissolving 20 mg of product in 650 µL of deuterated $CDCl_3$. The ^{1}H-NMR spectra were recorded at 298 K with a Bruker advance DRX 400 MHz spectrometer, in the Fourier transform mode.

Gel permeation chromatography (GPC). Apparent number-average molar mass (M_n), mass-average molar mass (M_w), and z-average molar mass (M_z), as well as the dispersity (Đ) of the PHOx were determined by GPC in chloroform at a temperature of 35 °C (flow rate of 1 mL/min) using an isocratic pump (VE 1122, Viscotek), a set of two PLgel 5 µm MIXED-C ultrahigh efficiency columns, a Shodex SE 61differential refractive index detector, and a variable wavelength UV detector (Spectra 100, Spectra-Physics).

Tensile tests. The mechanical properties of the samples were measured at 25°C on a Q800 dynamic mechanical analyzer (DMA) (TA Instruments) in DMA controlled force mode.

Rectangular samples of 25×5×0.5 mm were tested and a starting distance of 7 mm between the clamps was chosen. For each sample, an equilibrium of 5 min at 25 °C was first applied, before starting to apply a force with a ramp of 0.05 MPa/min at 25 °C until sample break. Samples were analyzed in dry state and in wet state (after immersion in MilliQ water for 24h). Strain hysteresis experiments were performed using the following method: equilibrium of 5 min at 25 °C, isothermal for 1 min, and measurement of the length of the sample, and then for each cycle: ramp stress of 0.02 MPa/min to 0.35 MPa, isothermal for 1 min, ramp stress of 0.02 MPa/min to 0.00 MPa, and isothermal for 5 min. This cycle was repeated 2 times (3 cycles in total). Data was analyzed with the TRIOS^{TM} (TA Instruments) software. The Young's modulus (E), the stress at break (σ) and the elongation at break (ε) of the samples were calculated by averaging reproductive values.

Equilibrium water absorption measurements (EWA). The equilibrium water absorption of PHOx was calculated by immersing each sample (previously weighed in dry state) in water at room temperature and weighting it after 24 hours (average of at least three values). EWA was determined by the following equation:

$$\text{Water swelling degree (EWA)} = (W_{s,w} - W_d)/W_d \times 100\%$$

where $W_{s,w}$ is the weight of the swollen sample in water and W_d the weight of the dried sample before swelling in water.

Water contact angle. The water contact angles were measured using a contact angle meter DGD Fast/60 and the software WINDROP by GBX Instruments in surface energy mode. For each measurement, a droplet of 15 µl of Milli-Q water was deposited on the surface of a PHOx film. Contact angles were then measured after 3 min and the measurements were repeated three times on three different spots to calculate an average value.

Differential scanning calorimetry (DSC). The presence of any phase-transition temperature and dehydration reaction were determined using a DSC 250 (TA Instruments) with a baseline flatness ≤10 μW with a precision of ± 0.01°C. The procedure was the following: equilibration at -80 °C, isothermal step for 5 min, and heating with a ramp of 5 °C/min up to 200 °C with signal modulation for 60 s and a modulation amplitude of ± 2°C. Thermograms were analyzed using the TRIOS^{TM} (TA Instruments) software.

Thermogravimetric analyses (TGA). Thermogravimetric analyses were performed on a TGA 2 model (Mettler Toledo) with a weight precision of 0.005% and a resolution of 1 μg. The procedure was the following: PHOx samples were placed under air atmosphere (20 mL/min) and underwent a first heating from 30 to 100 °C, with a heating rate of 20 °C/min. This was followed by an isothermal step at 100 °C for 10 min and finally a second heating from 100 up to 500 °C, with a thermal ramp of 10 °C/min. The curves were analyzed using the STARe software.

Rheological measurements. The complex viscosity of PHOx was determined at different temperatures by performing frequency sweep experiments on an ARES-G2 rheometer (TA Instruments) equipped with stainless steel parallel plate geometries (diameter of 25 mm). An interplate gap of 1 mm, a strain of 1% and a frequency scan from 0.1 to 100 Hz were set, while PHOx discs were placed between the plates so that the geometries were completely filled. A temperature sweep experiment (30–150 °C) was also performed on PHOx using a ramp of 2 °C/min. The same plates were used, with an interplate gap of 1 mm, a strain of 1% and a frequency of 1 Hz. Normal forces were monitored to be relaxed

before all measurements and the data was analyzed using the TRIOS^{TM} (TA Instruments) software.

PROCEDURES

Synthesis of the bis(α-alkylidene cyclic carbonate)

Bis(α-alkylidene cyclic carbonate) (4,12-dimethylene-1,3,9,11-tetraoxadispiro[4.2.4^{8}.2^{5}] tetradecane-2,10-dione, bisαCC) was synthesized by adapting Ouhib's protocol.[1] An ethynylmagnesium bromide solution (800 mL, 0.5 M in THF, 0.4 mol, 3.1 eq.) was first added in a 2 L two necked round-bottom flask under nitrogen atmosphere and concentrated by removal of 300 mL of THF under vacuum. 1,4-Cyclohexanedione (14.6 g, 0.13 mol, 1 eq.) was then dissolved in a minimal amount of dry THF before being transferred in a dropping funnel and added dropwise to the solution immersed in an ice bath. After stirring for 24 h at room temperature, the reaction was quenched by the addition of a saturated ammonium chloride (NH_4Cl) solution (260 mL). The formed precipitate was removed by filtration and diethylether (300 mL) was added to the filtrate. The aqueous phase was then extracted with diethylether (3 x 300 mL) and the combined organic phases were dried on $MgSO_4$, filtered, and dried under vacuum. The obtained 1,4-diethynylcyclohexane-1,4-diol was dissolved in diethylether (250 mL), purified by chromatography onto silica with diethylether as eluent and collected as a white solid after evaporation of the solvent (20 g, isolated yield = 94%). 1,4-diethynylcyclohexane-1,4-diol (20 g, 0.12 mol, 1 eq.), tetrabutylammonium phenolate (2 g, 6 mmol, 0.05 eq., previously synthesized following Grignard's protocol[2]), CuI (1.15 g, 6 mmol, 0.05 eq., previously purified by glacial acetic acid), and acetonitrile (40 mL) were added in a 250 ml high pressure autoclave. The reactor was heated at 40 °C and charged with a constant pressure of 100 bar of CO_2 for 24 h. After depressurization of the reactor, its content was

dissolved in CH_2Cl_2 (400 mL) and purified by chromatography onto silica (eluent: CH_2Cl_2). The solvent was then evaporated, and the obtained solid was dried under vacuum before being dissolved in acetonitrile (300 mL) and recrystallized at -20°C for 24 h. The solid was then filtrated, washed with cold acetonitrile, and dried under vacuum to obtain a white pure product (21 g, isolated yield = 69%). The bisαCC obtained was analyzed by ^{1}H-NMR (**Figure S1**). Structural characterizations are identical to those reported in the initial protocol and confirm the chemical structure of the product.

bisαCC: ^{1}H-NMR (400 MHz, $CDCl_3$) δ (ppm) = 4.82 (d, J = 4.3 Hz, 1H), 4.35 (d, J = 4.3 Hz, 1H), 2.09 – 1.97 (m, 4H).

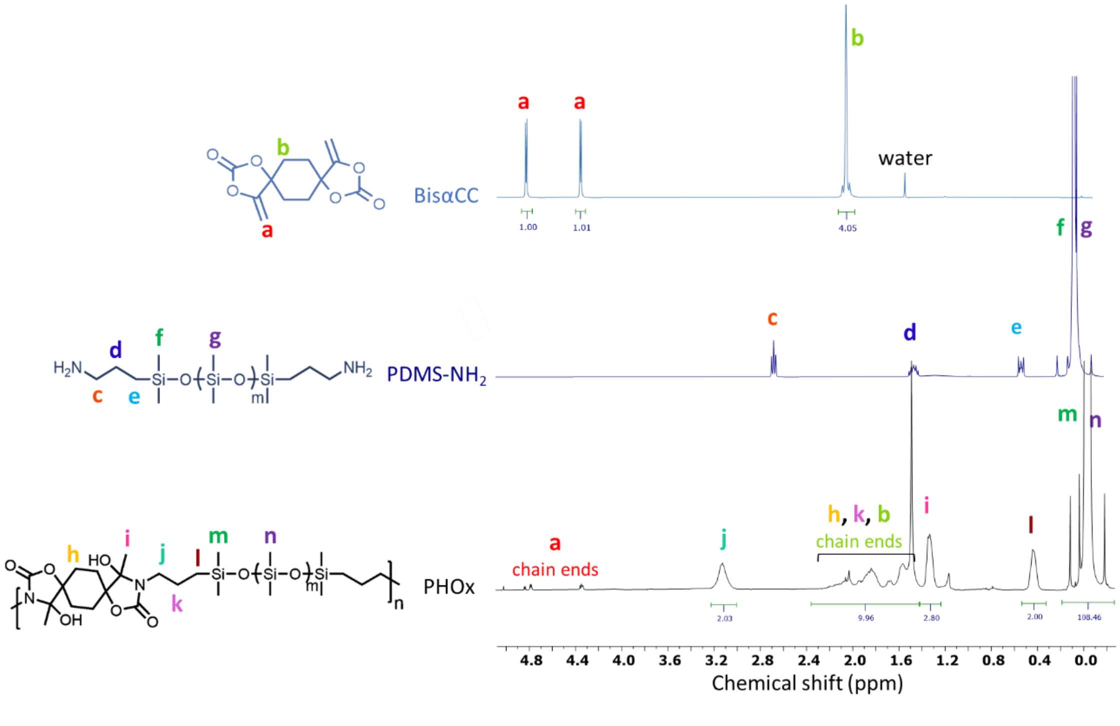

Figure S1. ^{1}H-NMR spectra of PHOx and its precursors (bisαCC and PDMS-NH_2 2,500 g/mol) (400 MHz, $CDCl_3$).

The ^{1}H-NMR spectra of PHOx and its precursors confirm the structure of the formed PHOx. Indeed, the peaks corresponding to the bisαCC monomer (a at 4.82 and 4.35 ppm and b at 2.09-1.97 ppm) as well as the peaks corresponding to the other precursor PDMS-

NH_2, namely c (2.69 ppm), d (1.53-1.43 ppm) and e (0.59 ppm), all disappear except for the chain-ends (visible in the PHOx spectrum), while the peaks corresponding to the PHOx are particularly visible at 3.13 ppm (j), 1.36 ppm (i) and 0.46 ppm (l).

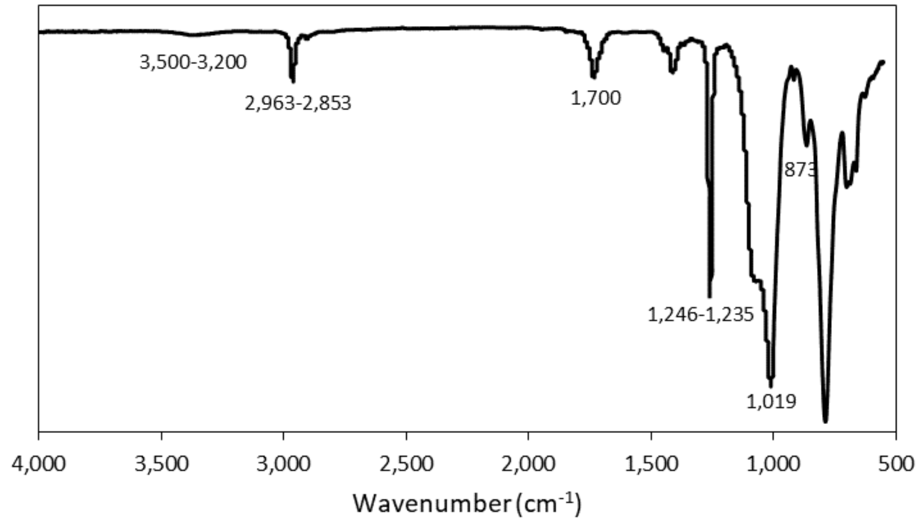

Figure S2. FTIR spectrum of PHOx.

The bands corresponding to the functional groups of PHOx are apparent, namely O-H and N-H stretching (3,500-3,200 cm^{-1}), C-H asymmetric and symmetric stretching (2,963 and 2,853 cm^{-1}), H-bonded C=O in urethane (1,700 cm^{-1}), and asymmetric stretching of N-CO-O as well as stretching of C-O-C (1,235 cm^{-1}), while the characteristic signals of its long PDMS segments are also present, such as Si-C (1,246 cm^{-1}), Si-O (1,019 cm^{-1}) and Si-C-H (873 cm^{-1}).

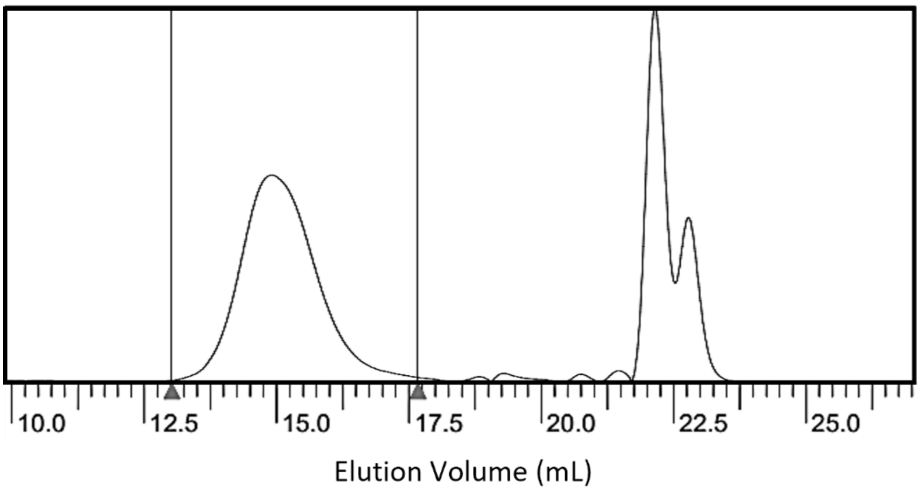

Figure S3. GPC chromatogram of PHOx in chloroform.

Apparent number-average molar mass (M_n) of 26,000 g/mol, mass-average molar mass of 58,900 g/mol (Mw), and z-average molar mass of 109,400 g/mol (Mz), as well as a dispersity (Đ) of 2.3 were determined by GPC in chloroform.

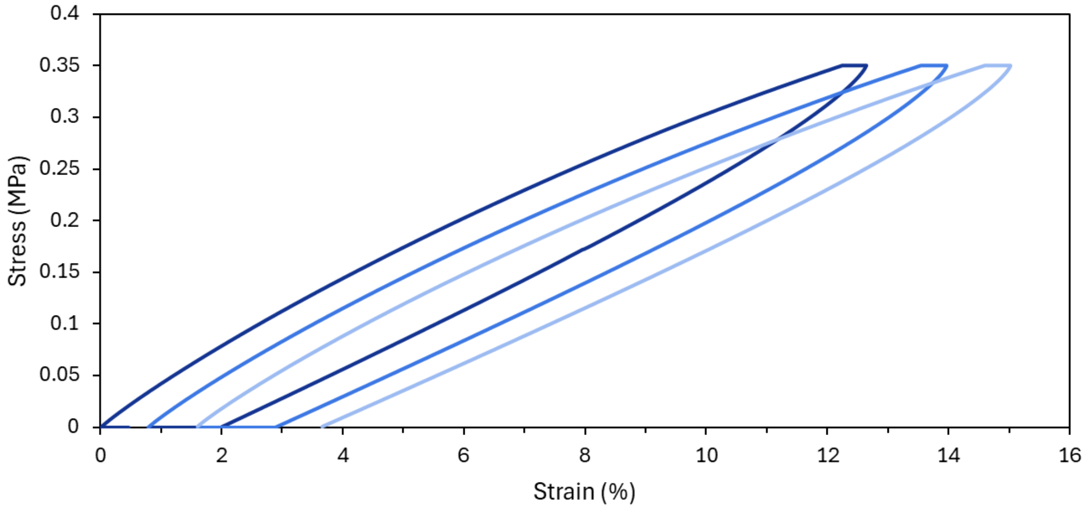

Figure S4. Strain hysteresis experiment performed on PHOx.

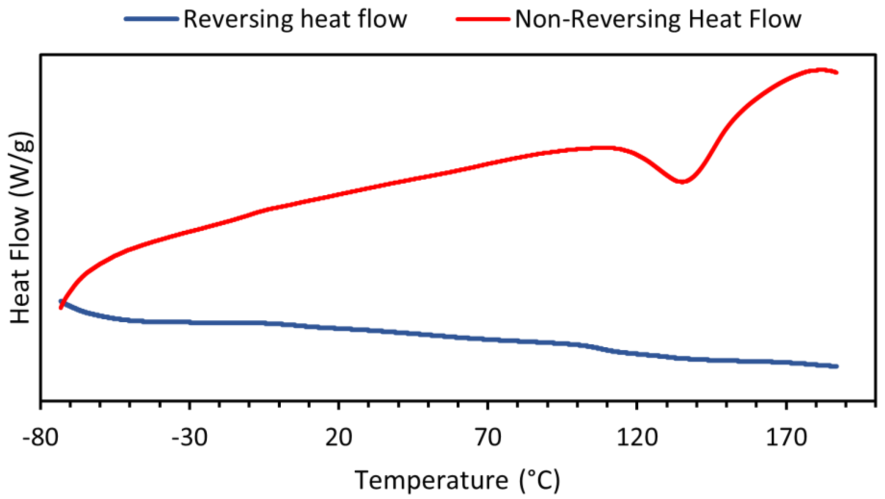

Figure S5. Modulated DSC curve of PHOx.

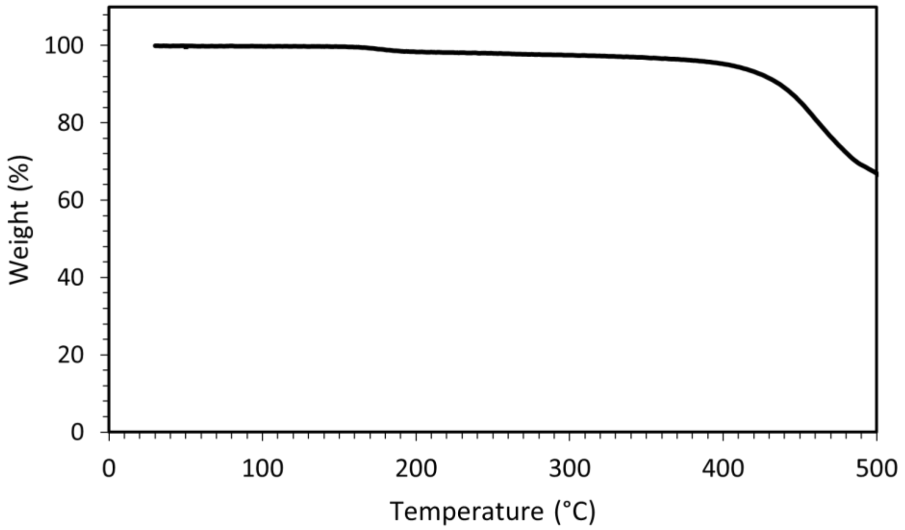

Figure S6. TGA curve of PHOx.

In vitro stability testing in physiological conditions

To test the stability of the new polymer in physiological-mimicking conditions, PU and PHOx discs were put in phosphate buffered saline (PBS) at 37 °C in an orbital shaker at 100 rpm for 8 weeks. Before the test, samples were weighed on their dry state and the pH of PBS was measured (pH meter accumet AE150, Fisher Scientific). Once a week, the discs were removed from the solution, the excess of PBS was wiped, and samples were weighed. Simultaneously, the pH of the test fluid was measured.

Table S1. Weekly pH measurements in the PBS solutions to evaluate PU/PHOx stability.

	W 1	W 2	W 3	W 4	W 5	W 6	W 7	W 8
PU	7.36 ±0.01	7.36 ±0.01	7.45 ±0.01	7.39 ±0.01	7.41 ±0.01	7.46 ±0.01	7.44 ±0.01	7.34 ±0.02
PHOx	7.32 ±0.01	7.39 ±0.01	7.47 ±0.01	7.37 ±0.02	7.42 ±0.03	7.47 ±0.01	7.41 ±0.01	7.31 ±0.01

Table S2. Weight of the samples (mg) measured weekly to evaluate PU/PHOx stability.

	W 1	W 2	W 3	W 4	W 5	W 6	W 7	W 8
PU	42.37 ±0.85	42.47 ±0.83	42.40 ±0.86	42.20 ±0.86	42.23 ±0.82	41.97 ±0.85	42.17 ±0.83	42.33 ±0.96
PHOx	47.73 ±0.39	47.57 ±0.26	47.57 ±0.37	47.37 ±0.29	47.37 ±0.40	47.33 ±0.31	47.23 ±0.25	47.40 ±0.36

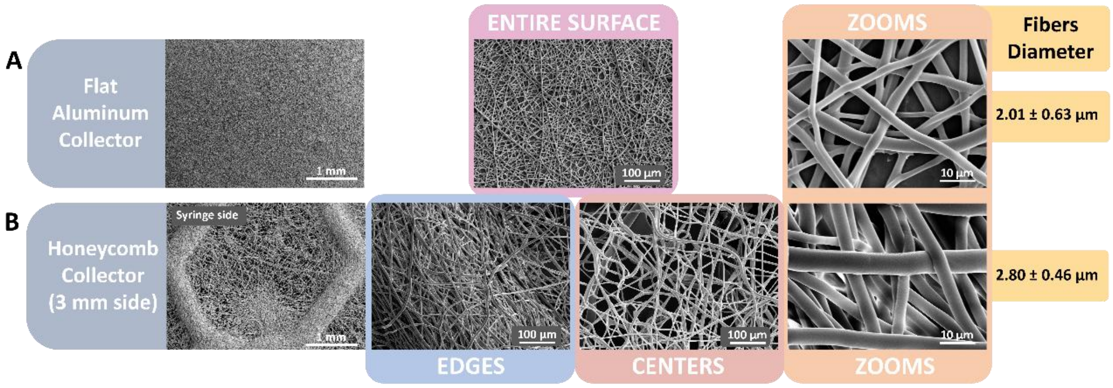

Figure S7. SEM images of the PHOx electrospun on a flat collector (aluminum sheet) (**A**), and on a honeycomb-patterned collector of 3 mm side (**B**).

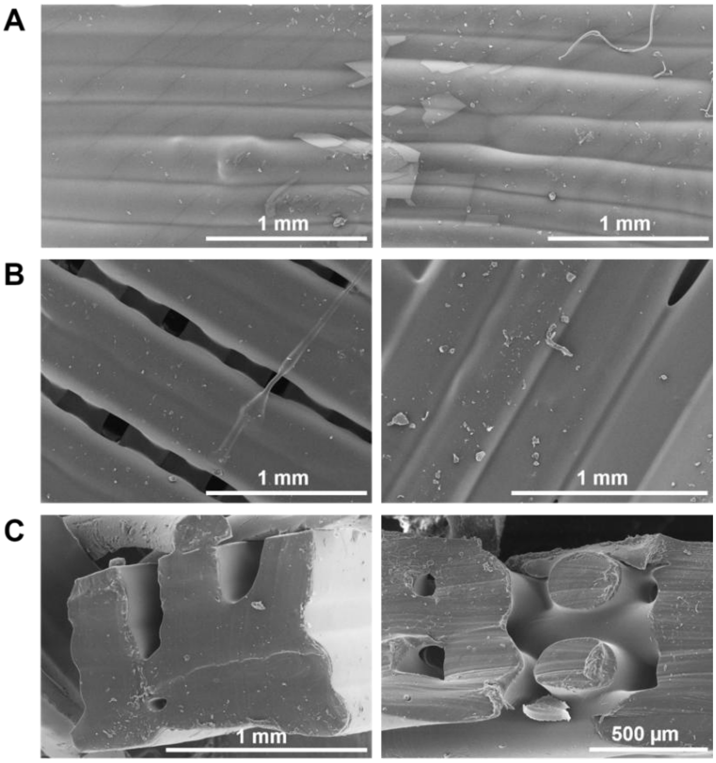

Figure S8. SEM images of the 3D printed PHOx by fused granule extrusion, taken in the Z (vertical) axis (**A**), X-Y (horizontal) axis (**B**), and cross-section (**C**).

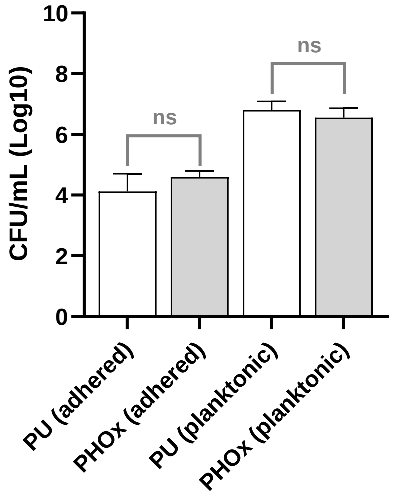

Figure S9. Quantification of the adherent and planktonic *S. epidermidis* after incubation with PU and PHOx discs for 24 h.

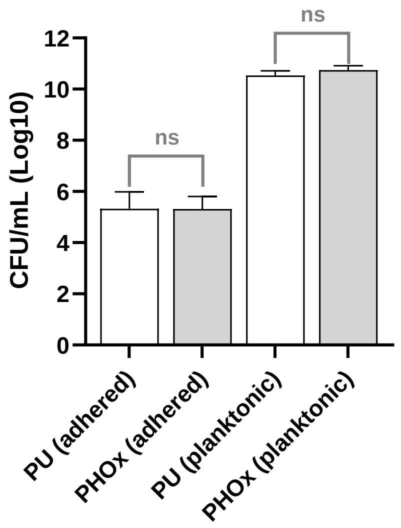

Figure S10. Quantification of the adherent and planktonic *P. aeruginosa* after incubation with PU and PHOx discs for 24 h.

Biological testing of the material

Hemolysis test (erythrocytes)

Red blood cells (RBCs) were incubated with PU and PHOx discs for 3 h at 37 °C in an orbital shaker at 100 rpm. Surface-area-to-volume ratios comply with ISO 10993-12, ISO 10993-4 and ASTM Standard F756-13. A solution of RBCs/dH_2O (1:7) was used as positive control (CTL +), while a solution of RBCs/PBS (1:7) was used as negative control (CTL -). After incubation, the solutions were centrifuged, and the absorbance of the supernatants was measured ($\lambda \approx 545$ nm). Absorbance values were compared to the CTL - and CTL +, and hemolysis rates were displayed in percentage.

Coagulation test (clotting time)

PU and PHOx discs were incubated with platelet-poor plasma (PPP) for 2 h at 37 °C in an orbital shaker at 100 rpm. PPP alone was used as CTL - of coagulation activation, while kaolin-coated glass coverslips were used as CTL +. After 2 h of incubation, human plasma clotting time was measured through a non-activated partial thromboplastin time (NaPTT) test.

Lactate dehydrogenase activity (platelet adhesion)

Briefly, PU and PHOx discs were incubated with platelet-rich plasma (PRP, 250 000 platelets/µL) for 2 h at 37 °C in an orbital shaker at 100 rpm. After 2 h of incubation, adherent platelets were washed with PBS and subsequently lysed with Triton (X-100) 1% v/v. Lactate dehydrogenase (LDH) activity in the lysates was measured every 5 min using a colorimetric assay kit (Sigma-Aldrich). Absorbance values ($\lambda \approx 450$ nm) were compared to positive and negative controls included in the assay kit. Through the analysis of a standard curve, the concentration of reduced nicotinamide adenine dinucleotide (NADH) in the samples was calculated.

Anti-adhesive properties against bacteria (24-h challenge)

Bacterial adhesion assays were performed using a biofilm-forming strain of *Staphylococcus epidermidis* (*S. epidermidis*) (ATCC 35984) and Gram - *Pseudomonas aeruginosa* (*P. aeruginosa*) (ATCC 15442). PU and PHOx discs were incubated with a

bacterial inoculum of 10^5 CFU/mL in tryptic soy broth (TSB) for 24 h at 37 °C under mild agitation (100 rpm). After the 24-h pre-adhesion step, polymer discs were rinsed three times with 500 µL of PBS. A multi-well plate containing the discs with adherent bacteria was then placed in a sonication bath for 30 min at room temperature with the nominal frequency of 50/60 Hz (sweep mode), to allow detachment of bacteria. Serial dilutions of detached bacterial suspensions were spot plated in tryptic soy agar (TSA) and CFUs were counted the next day. Similarly, 24-h planktonic bacteria (i.e. bacteria that did not adhere to the discs) were also quantified by spot plating serial dilutions of bacterial suspensions collected prior to rinsing the discs.

REFERENCES

1. Ouhib, F. *et al.* A Switchable Domino Process for the Construction of Novel CO2-Sourced Sulfur-Containing Building Blocks and Polymers. *Angew. Chem. Int. Ed.* **58 (34)**, 11768–11773 (2019).
2. Grignard, B. *et al.* Boosting the Catalytic Performance of Organic Salts for the Fast and Selective Synthesis of a -Alkylidene Cyclic Carbonates from Carbon Dioxide and Propargylic Alcohols. *ChemCatChem* **10**, 2584–2592 (2018).
